# …Fell Upas Sits, the Hydra-Tree of Death †, or the Phytotoxicity of Trees

**DOI:** 10.3390/molecules24081636

**Published:** 2019-04-25

**Authors:** Vadim G. Lebedev, Konstantin V. Krutovsky, Konstantin A. Shestibratov

**Affiliations:** 1 Forest Biotechnology Group, Branch of Shemyakin and Ovchinnikov Institute of Bioorganic Chemistry, Russian Academy of Sciences, 6 Prospect Nauki, Pushchino, 142290 Moscow, Russia; vglebedev@mail.ru (V.G.L.); schestibratov.k@yandex.ru (K.A.S.); 2 Department of Forest Genetics and Forest Tree Breeding, Georg-August University of Göttingen, Büsgenweg 2, 37077 Göttingen, Germany; 3 Laboratory of Population Genetics, Vavilov Institute of General Genetics, Russian Academy of Sciences, Gubkina Str. 3, 119991 Moscow, Russia; 4 Laboratory of Forest Genomics, Genome Research and Education Center, Institute of Fundamental Biology and Biotechnology, Siberian Federal University, 50a/2 Akademgorodok, 660036 Krasnoyarsk, Russia; 5 Department of Ecosystem Science and Management, Texas A&M University, 495 Horticulture Rd, College Station, TX 77843-2138, USA

**Keywords:** allelopathy, tree species, secondary methabolits, herbicidal activity, insecticidal activity, biopesticides, transgenic plants

## Abstract

The use of natural products that can serve as natural herbicides and insecticides is a promising direction because of their greater safety for humans and environment. Secondary metabolites of plants that are toxic to plants and insects—allelochemicals—can be used as such products. Woody plants can produce allelochemicals, but they are studied much less than herbaceous species. Meanwhile, there is a problem of interaction of woody species with neighboring plants in the process of introduction or invasion, co-cultivation with agricultural crops (agroforestry) or in plantation forestry (multiclonal or multispecies plantations). This review describes woody plants with the greatest allelopathic potential, allelochemicals derived from them, and the prospects for their use as biopesticides. In addition, the achievement of and the prospects for the use of biotechnology methods in relation to the allelopathy of woody plants are presented and discussed.

## 1. Introduction

Rice [1] defined allelopathy as the effect of one plant on growth of another plant through release of chemical compounds into the environment. In 1996, the International Allelopathy Society recommended the following, more wide definition of allelopathy: “any process involving the secondary metabolites produced by plants, microorganisms, viruses, and fungi that influence the growth and development of agricultural and biological system (excluding animals), including positive and negative effects” [2]. However, negative effects are observed more often than positive ones. Allelopathy plays a significant role in forest ecosystems for the following reasons: (1) trees release allelochemicals for long periods, and they may accumulate in soil to toxic levels, (2) one or only a few species dominate in the forest plantations, (3) allelochemicals affect the understory, and for some species they can affect the undergrowth, and in some cases can even cause problems in natural regeneration, and (4) introduced highly productive exotic trees may increase accumulation of allelochemicals in soil due to the inability of the local microflora to degrade them [3].

Allelochemicals are the products of secondary metabolism and have no nutritional value. According to the different structures and properties, allelochemicals can be classified into the 10 categories: (1) water-soluble organic acids, straight-chain alcohols, aliphatic aldehydes, and ketones; (2) simple unsaturated lactones; (3) long-chain fatty acids and polyacetylenes; (4) quinines (benzoquinone, anthraquinone, and complex quinines); (5) phenolics; (6) cinnamic acid and its derivatives; (7) coumarins; (8) flavonoids; (9) tannins; and (10) steroids and terpenoids (sesquiterpene lactones, diterpenes, and triterpenoids) [4]. Plant secondary metabolites include about 200,000 compounds, among which the most diverse are terpenoids (30,000), alkaloids (21,000), and phenolic compounds (8000) [5]. In allelochemical trees, the most common are the terpenoids and phenolics.

Reigosa [3] described five ways by which allelochemicals can be released into the environment from trees: stem flow, root exudation, leachates from aerial parts during rain, dew, and fog, volatiles from leaves or other plant parts, and litter decomposition. It is interesting to note that the greatest Russian poet Alexander Pushkin in his poem Anchar (1828) described all abovementioned ways of extracting poison from the *Antiaris toxicaria* tree except for litter decomposition. Studies have shown that various nutrients deficiency, as well as water stress, UV radiation, physical damage by herbivores or interspecific competition can enhance the production of allelochemicals and the sensitivity to allelochemicals [6].

For effective action, allelochemicals must reach a certain concentration. Their release is influenced by such environmental factors as temperature, duration and intensity of rainfall, type of soil and soil humidity [7]. Degradation by soil microorganisms also plays an important role in the accumulation of allelochemicals. Most allelochemicals are rapidly biodegraded in the soil, especially phenolics [7]. The term ‘‘phytotoxicity’’ was introduced to distinguish allelopathy (as interactions occurring in natural environment) from studies of plant extracts or allelochemicals (purified or synthetized) under controlled conditions [8]. The most common method for assessing phytotoxicity is the Petri dish bioassay of extracts (leachates) on seed germination and seedling growth. Such studies are usually carried out on model plants, for example, *Lactuca sativa*, because of their rapid germination and genetic homogeneity. It allows comparing the results among different studies [9]. Evaluation of plants in the soil, in the greenhouse, is rare and even less often in field conditions. Evaluation in natural conditions is especially important for the reason that allelochemicals mainly act through the soil. For a short period of time, a contact exposure through leachates during rain or dew may be possible. In some species, it may be conducted directly through volatile compounds. It was proved that some volatiles (e.g., terpenoids in the eucalypt species) can be dissolved and absorbed by soil [10]. This is especially important for woody plants which grow and accumulate allelochemicals in soil for a long period of time.

Various biological, chemical, and environmental factors can influence the allelochemical effectiveness for a particular species, such as: (1) specific plant toxins affect only particular species; (2) time or plant density required to reach toxic concentrations; (3) seasonal variation in toxicity; (4) synergistic effects of several allelochemicals; and (5) adsorption, leaching, and degradation in soils [11]. Coder and Warnell [12] described more than a hundred tree species with allelopathic activity. The strongest allelopathic effects were found in representatives of the genera *Acacia*, *Ailanthus*, *Eucalyptus*, *Juglans*, *Leucaena* and some conifer species. Most examples of allelopathy in trees are associated with exotic species that are rapidly becoming dominant in new ecosystems, such as Tree of Heaven (*Ailanthus altissima* (Mill.) Swingle), which is considered as one of the worst invasive plant species in Europe and also listed as an invasive plant in North America and many other countries [13]. At the global level, the invasion of alien organisms is considered the second largest threat to biodiversity after habitat destruction [14]. Nowadays, allelopathy is considered to be the most important factor influencing invasion and spread of exotic plants [15]. The ‘‘novel weapons hypothesis’’ [16] suggests that an introduced species may have strong allelopathic effects on neighbouring plant species in a novel environment, because native vegetation has not evolved resistance to unique allelochemicals produced by the invader. Rabotnov [17] hypothesized earlier that allelopathy is more significant between plants that have not co-evolved together for a long time. This hypothesis is especially important in forestry, because allelopathy could be one of the important factors influencing the successful introduction of tree species in new regions [18]. Some trees that belong to the widespread plantation species are also invasive, such as eucalypts. As a result, due to the large areas occupied by these trees and because of their dominance, the significance of allelopathy of woody plants can reach the level of ecosystems.

Agroforestry is an approach to the sustainable land management in which trees are being grown like agricultural crops or together with them. This combination of agriculture and forestry can promote biodiversity and reduced erosion, but tree allelopathy can have also a negative effect on understory growth. However, mixed plantations are often better than monocultural ones [19]. Still, some allelopathy problems may also arise even in these cases. Understanding the allelopathic mechanism can help to select the appropriate combinations of plants.

The effect of the trees’ allelochemics can vary from inhibiting to stimulating, which should be taken into account while selecting combinations of agricultural crops and trees for agroforestry systems. Autotoxicity is a type of intraspecific allelopathy, when plants release toxic chemicals into environment that inhibit germination and growth of the same plant species [20]. Autotoxicity is observed in woody plants in both natural forests and manipulated ecosystems, such as plantations. The ecological significance of autotoxicity lies in regulating plant population size over space and time, avoiding intraspecific competition and extending seed dispersal [20]. Under stress conditions, sensitivity to allelochemicals increases, and, thus, reducing the population size and, therefore, providing more resources to survivor plants [21].

Regeneration of natural forests is a very important ecological process in order to protect biodiversity and ecosystem balance. Autotoxicity is more common in coniferous forests, but some examples of it are also known for deciduous trees and forest plantations [20]. An important regulator of stand structure is the autotoxicity of tree allelochemicals [21]. The main reason for problems with natural regeneration in forest ecosystems is related to the litter, which falls intensively and can accumulate over years being the source of allelochemicals, particularly phenolics, which penetrate soil after degradation and leachation [20]. There are several potential mechanisms to avoid autotoxicity, including modification of allelochemicals, detoxification, sequestration, exudation, and resistance to the molecular target site, but yet there are no examples for the latter method available [22]. The most common methods are various postproduction modifications, such as glycosylation, methylation, and acylation, which not only reduce their toxicity by blocking reactive groups, but also increase stability and enhance water solubility, thus enabling transportation and/or storage in subcellular compartments [23]. The example of such protection in trees is the glycosylation of juglone [24]. Another method is the sequestering of toxins in vacuoles or in other specialized plant structures, such as trichomes or hairy glands [20]. This kind of protection is found in eucalypts: monoterpenes occur in secretory trichomes, and then they are released after volatilization. The detoxication of its own allelochemical is a relatively rare case. It is assumed that it is present in the seedlings of *Leucaena leucocephala*, which detoxifies mimosine into 3,4-dihydroxypyridine and then converts it into non-toxic metabolites [25]. The way of tree protection, when tree secrets a compound almost as quickly as it is produced, has also not yet been described. In addition, some inactive allelochemicals released by plants can be converted into active forms after degradation, for example, by soil microbes in some cases [20] or environmental factors (temperature, light, oxygen, etc.), for example, the toxic juglone is produced by oxidation of the non-toxic hydrojuglone.

Plants can vary significantly in their sensitivity to the same type of phytotoxin. It has been shown that herbaceous species in the *Ericaceae* and *Aquifoliaceae* families are very sensitive to the black walnut, while the members of *Liliaceae*, *Malvacea*, and *Taxaceae* are highly tolerant, but the mechanism of tolerance to juglone is currently unknown [26]. The mechanism of this stability is unknown, but Orcutt and Nilsen [27] suggested the following protection options: (1) uptake reduction of allelochemicals at the surface of root, (2) compartmentalization of allelochemicals away from molecular target sites, and (3) detoxification of allelochemicals. It is known that plants that have been growing for a long time near the allelopathic species usually have a certain resistance. The study of these plants will help to understand the mechanisms of their protection.

Plants are responding dynamically to changes in the environment through rapid induction and reversal of secondary metabolite production [28]. Terpenoids and phenolics in leaves and terpenoids in emissions are the secondary metabolites of forest trees most frequently studied in the context of their response to climate change [5]. The main changes are temperature increase and CO_2_ content. Studies have shown that higher concentration of CO_2_ increases phenolics in foliage and in emissions, while warming has the opposite effect. In addition, CO_2_ together with warming increased phenolics in foliage, but reduced phenolics in woody tissues [5]. It should be taken into account that climate change can also lead to changes in allelopathic activity. This fact is especially important for woody plants that have been cultivated in the same place for many years.

## 2. Allelopathic Tree Species

### 2.1. Ailanthus Altissima

Tree of Heaven (*Ailanthus altissima* (Mill.) Swingle) from the *Simaroubaceae* family is a deciduous tree native to northeastern and central China. Due to its decorative properties, *Ailanthus* was introduced in Europe in the 1740s and the USA in the 1780s and has since spread widely across all continents except Antarctica [29]. *Ailanthus* is very fast growing, possibly the fastest growing tree in North America [30]. It possesses high fecundity—it is able to multiply as seeds, up to 300,000 units per season from a tree—which extend up to 200 m followed by 100% germination, and can propagate vegetatively from stumps and roots [31]. In addition, *Ailanthus* is highly resistant to pollutants and able to grow on poor soils, and contains allelochemicals [32]. All these factors make it highly invasive, especially in areas that are disturbed by human activity, such as roadsides and railways, wastelands and deforestation, and only cold and shading can limit its appearance and spread [13]. The negative effect of *Ailanthus* on the growth of neighboring plants was noticed a long time ago. For the first time, its phytotoxicity was evaluated by Mergen [33], who in a greenhouse experiment found a reduction in the growth of seedlings of 35 species of gymnosperms and 10 species of angiosperms after treatment with *Ailanthus* leaflet extract, and only *Fraxinus americana* L. plants were not affected. Subsequently, the inhibitory effect of root bark powder was shown on *Lepidium sativum* L. [34], and Lawrence et al. [35] demonstrated that the extracts of the stem and leaves of *A. altissima* inhibit the germination and growth of seedlings of both the test species *Lactuca sativa* and those growing near *Ailanthus*—six herbaceous and one woody (*Platanus occidentalis*) species. Water extracts of *A. altissima* inhibited the germination and growth of radish seeds (*Raphanus sativus* L.), watercress (*Lepidium sativum* L.), portulaca (*Portulaca olearacea* L.) [32], oats (*Avena sativa* L.), rapeseed (*Brassica napus* subsp. *Oleifera*), and sunflower (*Helianthus annuus* L.) [36]. Leaf methanol extracts also inhibited germination and growth of *Daucus carota* (carrot) roots depending on the concentration [37]. In addition to higher plants, extracts of *A. altissima* exerted an inhibitory effect in a dose-dependent manner on the cell density of the cyanobacterium *Microcystis aeruginosa* and reduced the number of extracellular cyanotoxin microcystins [38]. In addition, greenhouse studies showed that the soil from *Ailanthus* stands significantly inhibited germination and growth of the local species *Verbesina occidentalis*, but did not affect the invasive in North America species *Dipsacus fullonum* [39]. The difference may be related to species specificity, but the authors suggest that *A. altissima* may affect native and non-native species in different ways, potentially promoting the spread of other non-native plants in the invasion affected community.

These experiments were carried out under laboratory and greenhouse conditions, but they have a number of drawbacks that can be overcome only by field tests, which allow to test the following: (1) accumulation and preservation of allelochemicals in soil, (2) toxic effects on coexisting species, and (3) change in allelochemical effects in space and time [40]. Such studies are especially important for long-lived woody species, but they are quite rare. For the first time, similar studies were conducted by Gomez-Aparicio and Canham [40], which for two years have been evaluating the effect of *Ailanthus* on germination and growth of seedlings of three native tree species (*Acer rubrum, A. saccharum*, and *Quercus rubra*) in temperate forests of the United States. The effect was negative and species-specific. It was apparently influenced by both the differences between species, the response to allelopathy and changes in the availability of resources caused by the presence of *Ailanthus*. *A. altissima* significantly impoverished understory vegetation in the suburban forest of Fontainebleau in Paris area. There was a significant negative correlation of floristic richness with root suckers, which means that *A. altissima* allelopathy can be found in the area [41].

### 2.2. Eucalyptus

*Eucalyptus* trees (Myrtaceae) are native in Australia and New Zealand and one of the fastest growing and highly productive trees in tropics and subtropics. They have been introduced into more than 120 countries and comprise one-third of the world’s total plantation area [42]. The allelopathic properties of eucalypts have long been known. Lerner and Evenari [43] showed in their laboratory experiments that leaf extracts of *Eucalyptus rostrata* inhibited seed germination. Natural fog drip from *E. globulus* inhibits the growth of annual grass seedlings in bioassays [44]. Since then, the impact of different *Eucalyptus* species was investigated in bioassays on different types of weeds, crops and tree species. May and Ash [45] showed that eucalypt extracts affected germination of the *Lolium* species. They came to the conclusion that allelopathy was likely to be a cause of the understory suppression by the *Eucalyptus* species. Aqueous leachate of fresh leaves of *E. globulus* showed an inhibitory effect on two perennial weeds: *Cyperus rotundus* L. and *Cynodon dactylon* L. Pers. [46]. Studies in the greenhouse confirmed that the leaf litter extract of *E. camaldulensis* had an inhibitory effect on *Vigna unguiculata*, *Cicer arietinum*, and *Cajanus cajan*. Moreover, it inhibited the latter species to a greater degree. Therefore, eucalypt-based systems are not recommended for use in agroforestry [47]. The influence of the aqueous leaf leachate and leaf volatile of *Eucalyptus urophylla* on seed germination and seedling growth of seven native and three exotic tree species was studied by Fang [48]. The allelochemical effects varied depending on the dose and tested species. Aqueous and ethanolic extracts of *E. erythrocorys* L. caused an inhibitory effect on both weeds (*Sinapis arvensis* L. and *Phalaris canariensis* L.) and a cultivated crop (*Triticum durum* L.). Seedling growth was a more sensitive indicator than seed germination [49]. Finally, the eucalypt allelopathic properties were investigated to control algae bloom, which degrades water quality by producing the most potent toxins, which is a serious threat. Zhao et al. [50] was the first to study the effects of leaf extracts of *Eucalyptus grandis* × *E. urophylla* hybrids on the density of algae cells in mesocosm. Direct planting of eucalypts was significantly more effective, since, in addition to the isolation of allelochemicals, competitive absorption for macronutrients occurred as well. At the same time, plants or extracts had no adverse effect on diversity or abundance of the microbial community [50].

Due to the longevity of woody plants, it can be assumed that their allelochemical properties may change over time. To test this assumption on the three species (*Raphanus sativus*, *Phaseolus aureus*, and *Lolium perenne*), aqueous root extracts and rhizosphere soil from *E. grandis* plantations of different ages (2-, 4-, 6-, 8-, and 10-year old) were evaluated [51]. The extracts inhibited the germination and growth of plants, and the younger ones were affected to a larger degree. On the other hand, soil samples from 6, 8, and 10-year-old plantations demonstrated a remarkable stimulative effect on *L. perenne*. Thus, when evaluating the allelopathy of woody plants, their age should be taken into account.

Experiments for plant allelopathy are often evaluated at the morphological (assessment of germination and growth) and physiological levels (photosynthesis, respiration, and enzyme activity). The eucalypt extracts or leachates demonstrated various effects at the physiological level, such as a decrease in the respiration rate and protein, carbohydrates, and nucleic acid contents [52], a change of activity of various antioxidant enzymes [53], increased H_2_O_2_ levels, and electrolyte leakage of the seedling membranes [54]. Such reactions, in general, were observed also for allelochemicals of other plants. The cytotoxic and genotoxic effects were much less evaluated. Under the influence of leaf extracts of *E. globulus* the *Hordeum vulgare L.* plants demonstrated significant mitotic abnormalities such as disturbed metaphases and anaphases and chromatin bridge [55]. In another survey, the comet assay and semi-quantitative RT-PCR methods were used to assess the genotoxic impact of the *E. globulus* leaves on soybean genome [56]. The assay showed a steady increase in the frequency of DNA damage in soybean nuclei and changes in transcript amounts of cysteine proteases and specific inhibitors genes. This research showed that *E. globulus* allelochemicals can have variable genetic effects on soybean plant and ultimately caused growth delay and yield falling [56].

Due to the fact that eucalypts are of great economic importance and occupy very large areas, field experiments with eucalypts are carried out more often than with other allelopathic trees. In allelopathic studies, the main object of study is leaves or litter. Meanwhile, it is widely known that the continuous planting of eucalypts in monoculture can cause the accumulation of phytotoxins in soil, which leads to its degradation and loss of productivity [57]. The allelopathic effect of leaf litter and living roots of *E. urophylla* on seed germination and seedling survivorship of three common native tree species—*Delonix regia*, *Tsoongiodendron odorum* and *Elaeocarpus sylvestris*—were studied in [58]. These field experiments showed that the presence of the *E. urophylla* roots significantly inhibited the growth of seedlings of all three species, unlike the litter which did not affect them. Later, this experiment was extended to 12 native broad-leaved tree species. All of them were endemic for South China and had high economic value. The experiment lasted for more than two years [59]. The results showed that poor establishment of native trees in the *Eucalyptus* plantations were mainly due to the *Eucalyptus* roots rather than its litter. On the opposite, the litter stimulated the germination and growth of seedlings of the most tested species. However, an experiment with crops gave another result. Zhang et al. [60] evaluated the effect of three eucalypt species (*E. urophylla* Blake, *E. citriodora* Hook., and *E. camaldulensis* Dehnh) on *Raphanus raphanistrum* var. *sativus* L. G. Beck, *Cucumis sativus* L., and *Brassica rapa* var. *glabra* Regel, but instead of living roots, live root exudates were used. It was found that the allelopathic effects of the leaf litter extracts were stronger than the root exudates. According to the results, it was concluded that radish is unsuitable for cultivation with these species of eucalypts, while cucumber and Chinese cabbage can be cultivated provided that the leaf litter is removed. Although, Chinese cabbage could be grown only with *E. urophylla*. The effect of leaf litter of *E. camaldulensis* depended on the species [61], when a leaf litter was added to the soil in doses of 100 to 2000 kg/ha with an average accumulation of 1028 kg/ha/year litterfall in a plantation. The litter had no effect on growth of crops *Vigna unguiculata, Cicer arietinum*, and *Cajanus cajan*, but significantly reduced it in woody plants *Leucaena leucochephala* and *Albizia procera*. The litter inhibited root growth to a greater extent than shoot growth. There was an inhibitory effect on nodulation of *Vigna unguiculata* and *Albizia procera*. This study confirmed the allelopathic effects of *E. camaldulensis* in field conditions, where the influence of numerous biotic and abiotic factors have been taken into account along with a careful selection of crops for agroforestry systems [61]. The results of this experiment are somewhat different from the studies in the greenhouse, where it showed that for mixed plantations with *E. camaldulensis* presence it is better to use *Leucaena leucochephala* than *Albizia procera* [47].

The discrepancy between laboratory and field experiment results was shown in studies of *Eucalyptus saligna*. In the laboratory, the aqueous extract of the *E. saligna* leaf litter severely reduced germination and growth of graminoid and forb species [54]. However, the field studies demonstrated that inhibitory effects of the *E. saligna* leaf litter on the establishment of grassland species *Paspalum notatum* (Poaceae) and *Lotus corniculatus* (Fabaceae) were not related to allelopathy but were mainly associated with the physical effects of litter [62]. This once again confirms the importance of field studies of allelopathic interactions.

Forest plantations, as a rule, are established using the same species, but they are less resistant to pests and diseases and can harm environment due to soil degradation and loss of biodiversity. Therefore, in recent years, interest in mixed plantations, which are devoid of these shortcomings, has increased. Moreover, it has been shown that mixed-species plantations of *Eucalyptus* with an N-fixing species possess increased productivity compared with *Eucalyptus* monocultures [57]. However, before laying out such plantations, it is necessary to assess the allelopathic compatibility of the species.

Field tests with four common broad-leaved tree species, three native subtropical evergreen forest species *Acmena acuminatissima, Pterospermum lanceaefolium*, and *Cryptocarya concinna*, and one introduced nitrogen-fixing species *Albizia lebbeck*, showed significant differences between plantations of *Eucalyptus urophylla* and *Pinus elliottii* [63]. The root growth of the three forest species sown in the soil where the eucalypt plantation was located was significantly reduced in comparison to the pine plantation, but no difference was found for *Albizia lebbeck*. The authors believed that these differences were not necessarily related to the status of plants (native or introduced), but most likely with responses of N-fixing and non N-fixing trees to allelopathy [63]. The experimental results suggested that the N-fixing trees can be used in the mixed with eucalypt plantations. However, these results were not consistent with another extensive study, where in order to evaluate allelopathy in the field conditions, *E. urophylla* was planted among the seedlings of 20 broad-leaved woody species, and their survival and growth rate were evaluated during the 10-year period [64]. Based on the results of this experiment 20 species could be divided into two types: inhibited and stimulated/unaffected by aqueous extracts of *E. urophylla*. Compared to the inhibited species, the uninhibited species grew faster and survived better. In this study, the three N-fixing species did not show better survival and growth than non-N-fixing species, while *A. lebbeck* and *L. leucocephala* demonstrated the lowest survival rate and growth rate. This study allowed to select woody species that are good candidates for the mixed with *E. urophylla* plantations [64]. In addition, this 10-year field experiment led to the conclusion that the reduction in plant biodiversity in *E. urophylla* plantations was more associated with allelopathy than with competition for resources.

### 2.3. Fabaceae

*Acacia dealbata Link*, originated from Australia, is one of the most invasive plants in many parts of the world, where its expansion leads to a decline in populations of native species and threatens local plant biodiversity [65]. This species was introduced into Europe as an ornamental in the 1790s and quickly became invasive in the Mediterranean countries due to its rapid growth, high fertility and fast resprouting from stumps and roots following cutting, fire or frost [66].

Involvement of the *A. dealbata* allelopathy in the competition was demonstrated by Reigosa et al. [67], who proved that soil extracts from the *A. dealbata* sites during its flowering inhibit germination and growth of *Trifolium repens* and *Lolium perenne*. It was later shown that soil percolates from the *A. dealbata* plantation inhibited the germination and growth of *Lactuca sativa* stronger than throughfall and stemflow, and an increase in phytotoxicity coincided with the acacia flowering period and the germination of undergrowth species, which increased the allelopathic effect [68]. Lorenzo et al. [69] evaluated the role of allelopathy in the distribution of *A. dealbata* in Southern Europe using throughfall, litter leachate, and aqueous soil extracts collected in the main phenological and stress phases of the tree, i.e., formation of pods, in a period of severe drought, during formation of inflorescences, and in the flowering period. The effects of the *A. dealbata* extracts on the germination and growth of *Lactuca sativa*, *Arabidopsis thaliana*, *Zea mays*, and *Dactylis glomerata* were species-specific and more often stimulating rather than inhibiting. Such a reaction can have long-term negative effects on native plant populations, since advanced growth during periods of scarce resources can be harmful [69]. The decomposition of the *A. dealbata* plant materials showed high phytotoxic activity against *Lactuca sativa*, *Trifolium repens*, and *Lolium perenne*, with toxicity being maintained for up to 16 weeks [70]. In order to investigate the mechanism of germination and growth inhibition, evaluation of the physiological parameters of the native understory species in Northwest Spain was carried out [71]. Net photosynthetic and respiration rates of *Hedera hibernica*, *Dicranum* sp., *Dactylis glomerata* L., and *Leucobryum *sp. were significantly affected by canopy leachate and apical branche macerate of *A. dealbata*, with the strongest effect during the flowering period. These results showed that *A. dealbata* could limit distribution of understory species [71].

In order to obtain a solution of the *A. dealbata* allelochemicals at natural concentrations Aguilera et al. [72] factored in amount of precipitations and naturally accumulated litter in Chile under conditions similar to the Mediterranean climate. The obtained aqueous extracts of various parts of *A. dealbata* showed a significant inhibition of growth, but not germinating capacity of *Lactuca sativa* L. Since all parts of the plant induced inhibition, *A. dealbata* is capable of showing allelopathic properties during the entire phenological cycle.

It is considered that the invasive properties of *A. dealbata* are also caused by allelopathy, but the importance of this factor among others (i.e., direct competition, changing growth conditions and soil properties) has not been tested. The study in natural environment [73] suggested that the main factor affecting the establishment of both native and invasive species is shaded microhabitat in dense populations of *A. dealbata*, rather than changes in soil properties or allelopathy. The authors concluded that a negligible effect of *A. dealbata* allelopathy implies a secondary role of allelochemicals during the invasion of this species in European forests. The results of this research contradicted the previous ones that showed a significant allelopathic effect on various plant species, which proves yet again the importance of field studies.

Studies on allelopathy were also carried out on other types of acacia. Extracts of phyllodes from *Acacia melanoqlon R.Br.* inhibited growth of *Lactuca sativa* rather than its germination [74], and flower extracts proved more phytotoxic than phyllodes against germination and growth of *Dactylis glomerata*, *Lolium perenne*, *Rumex acetosa*, and *Lactuca sativa* [75]. The *A. mangium* acueous leaf extracts inhibited germination and growth of *Oryza sativa* [76]. For the first time, the autotoxicity of *Acacia* species has been shown in Noumi et al. [77], where the aqueous extract of the *Acacia tortilis* leaves in high concentrations significantly inhibited seed germination. At the same time, positive autoallopathy was shown for *A. dealbata*: the root growth was stimulated with its own extracts [69]. One of the very few field studies on allelopathy in acacias was carried out on *A. pennatula* in Nicaragua. Seedling survival of native tree species *Guazuma ulmifolia Lam*., *Enterolobium cyclocarpum Griseb*., and *Cedrela odorata* L. under the canopy was about 20–30% lower than outside [78]. Seedling mortality increased with the advance of the dry season, although higher soil moisture conditions were under the canopy. The authors believed that *A. pennatula* inhibits the growth of plants under the canopy with the help of allelopathy, reducing the relative weight ratio of roots, which is critical during the dry season.

There is another tree plant with allelopathic properties in the family of Fabaceae—*Leucaena leucocephala*. This fast-growing nitrogen-fixing tree, native in Mexico and Central America, is notable for its tolerance to various abiotic and biotic stresses. Its introduction as an animal fodder began in the 16th century from the Philippines, and since then it has spread widely across tropical and subtropical regions [79]. Due to its high nutritional value—the concentration of crude protein comprises about 30%—*Leucaena* is known as the “alfalfa of tropics” [80]. However, high contents of anti-nutritional factors, such as mimosine and condensed tannin, can lead to a number of toxic symptoms in animals and limits nutritive value of its foliade [81]. The leaves and litter of *L. leucocephala* inhibited germination and growth of *Zea mays* in laboratory and greenhouse studies [82]. It was shown that phenols (a well-known group of allelochemicals) in the leaves and litter were responsible for the allelopathic effect. Evaluation of the influence of fallen leaves of *L. leucocephala* on a tree species *Albizia procrai* and three crops *Vigna unguiculata*, *Cicer arietinum*, and *Cajanus cajan* showed that litter stimulated shoot growth in low doses and inhibited it in high doses [47]. The phytotoxicity of *Leucaena* against aquatic weeds has also been tested. Leaf disc assay with *Eichhornia crassipes* (Mart.) Solms., an invasive aquatic weed in many regions of the world, showed that leaf leachate of *L. leucocephala* enhanced electrolyte leakage, decline mitochondrial respiration and inhibits antioxidant enzymes in treated tissues [83].

Species from the family Fabaceae have a great advantage compared to other allelopathic trees. Being nitrogen-fixing plants, they contribute to the increase of nitrogen in the soil. A number of studies have shown that acacia and *L. leucocephala* improve the nitrogen status in soils of both mixed forest plantations and agroforestry systems [84]. Thus, co-cultivation of agricultural or forest plants together with nitrogen-fixing species with positive allelopathy will help to increase their productivity, provided they are correctly selected.

### 2.4. Juglandaceae

Walnut phytotoxicity is the oldest reported example of allelopathy. Roman naturalist and natural philosopher Pliny the Elder was the first to describe its inhibitory effect on surrounding vegetation in his *Naturalis Historia* (circa 77 AD) stating that “the shadow of walnut trees is poison to all plants within its compass” [85]. It was found later that other plants of the *Juglandaceae* family, such as black walnut (*Juglans nigra* L.), also possess this ability. These plants’ allelopathy under the laboratory conditions was demonstrated almost 100 years ago, when Massey [86] discovered that the black walnut root bark extract caused wilting of tomato plants.

Unlike forest tree species with allelopathic properties nuciferous are considered to be agricultural crops; and any possibility of their cultivation together with other cultivated species possesses considerable interest. In order to select species that could be cultivated in vicinity of a walnut tree, Kocacaliskan and Terzi [87] evaluated the walnut leaf extracts effect on germination and growth of 11 agricultural crops, both monocotyledons and dicotyledonous. The effect depended on the species, and the growth was inhibited to a greater extent than germination. It was unexpectedly demonstrated that the growth of *Cucumis melo* was stimulated by the extract, which indicates a possibility of its cultivation near the walnut trees. Experiments conducted in a greenhouse showed that walnut extracts were strongly inhibiting both vegetative and reproductive growth of *Fragaria×ananassa L.* plants, and were reducing the N, K, Ca, Fe, and Mn content in leaves, as well as total soluble solid and vitamin C in berries [88]. Allelochemicals could not only be washed out of leaves by rain, but also fall into soil from leaf litter. To assess allelopathic activity of the walnut leaf litter, it was added to the soil for cultivating the *Lactuca sativa* var. *angustata* [89]. Tree waste was inhibiting the shoot weight of lettuce especially during the early growth stage or with large amounts, while also reducing the chlorophyll *a* and *b* and carotenoids content. Twenty-eight compounds were identified with three solvent extracts, and some of them, such as lupenone, lupeol, fatty acids, and phenolic acids possess the allelopathic activity [89].

In addition, the walnut extracts efficiency was tested on weeds. NatureCur^®^ preparation based on the extract from leaves, fruits, and branches of black walnut was evaluated on weeds in laboratory (four species), greenhouse (seven species), and in a commercial almond orchard that was naturally infested with *Conyza canadensis* [90]. The preparation was inhibiting seed germination in Petri dishes and was causing complete loss when being treated with 20–43% concentration depending on the type in a greenhouse and with a 43% concentration when being treated under field conditions. The extract showed properties of systemic herbicide with xylem transport and had prospects as a pre-and post-emergence bioherbicide.

### 2.5. Other Decidious Trees

Studies of other hardwood angiosperm tree species and their allelopathic properties carried out since 2000 are presented in Table 1.

Some of them have already been mentioned in the list of trees with allelopathic activity compiled by Coder and Warnell [12], the phytotoxicity of others has been studied only recently. As a rule, these are tropical and subtropical species, some of which are used in ethnopharmacology. For example, it was shown that leaf extracts of the evergreen tree *Alstonia scholaris* from South and Southeast Asia have herbicidal activity against *Parthenium hysterophorus* L. [94]. Extracts of leaves of *Rhododendron formosanum*, an endemic species in the Taiwan Mountains, contains various phenolic phytotoxins and inhibited plant growth in greenhouse and laboratory experiments suggesting that it is associated with allelopathy [124].

### 2.6. Gymnosperm Species

Conifers differ from the angiosperm species mentioned earlier in the article. The latter ones were invasive, plantation or agricultural trees that are not threatened with a reduction in habitat and which usually use allelopathy against other species, then there is a different situation with conifers: as many natural coniferous forests are cut down due to their high commercial value, but their restoration is difficult. Unfortunately, natural regeneration doesn’t occur at all or is very slow. Coniferous forests are usually characterized by lesser understorey vegetation and by regeneration problems, and one of the reasons for this is allelopathy, although other differences from angiosperms such as slower growth, later fruition, etc., also play a role. Allelopathy in conifers often manifests itself in the form of autotoxicity, and this has been reported for different conifer species [133].

Apparently, the first observation about conifer congestion allelopathy appeared more than 300 years ago in Japan: Lee and Monsi [134] found an ancient document in which Banzan Kumazawa reported that red pine (*Pinus densiflora* Sieb. Et Zucc.) was harmful to crops growing under their canopy. More than 100 species are included in the *Pinus genus*; and they are widely spread as native species throughout the entire Northern Hemisphere occupying large areas in South America, Australia, and New Zealand as plantation species. Understory vegetation under the canopy of pine trees in several species is rather poor, and this is not associated with shading, since pine forests are characterized by high intensity sunlight. Allelopathic interactions are assumed to play an important role under these conditions [1]. Much research on coniferous allelopathy is associated with various types of pine trees, possibly due to the great economic value of these plants. Allelopathic species of conifers are characterized by a large variety of allelochemicals, and therefore will be discussed in detail in the relevant chapter. Species with allelopathic activity studied since 2000 are presented in Table 2.

## 3. Allelochemicals in Trees

### 3.1. Ailanthone

The tissues of various *Ailanthus* species contain a large number of biologically active compounds, such as alkaloids, terpenoids, steroids, flavonoids, and volatile oils—about 200 in total [37]. Studies periodically discover new ones, for example, in the *A. altissima* bark [161] or fruits [162]. Among them, the quassinoids stand out and represent highly oxygenated degraded triterpenes with a bitter taste, which can be found only in the *Simaroubaceae* family. They take their name from the first compound of this class—quassin—isolated from *Quassia amar* [163]. The bioactivity of quassinoids is based on plasma membrane NADH oxidase inhibition [164].

**Figure 1:**
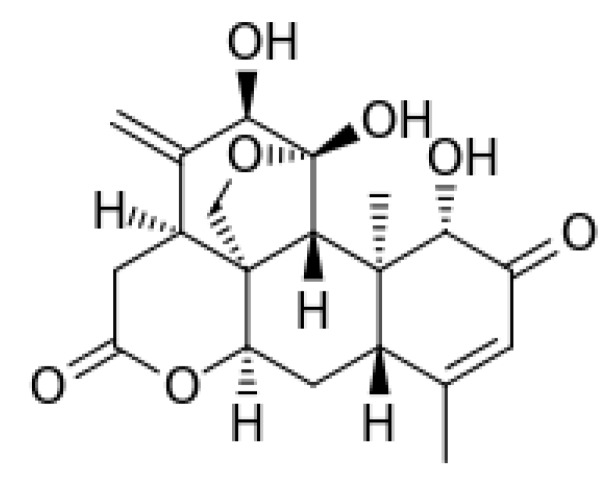
Ailanthone.

**Figure 2:**
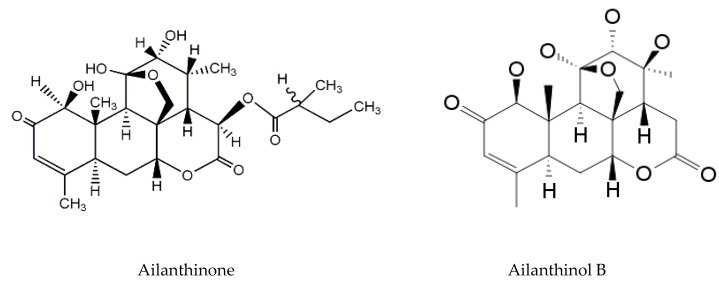
Allelochemicals of *Ailanthus altissima*.

*A. altissima* produces a range of quassinoids, including ailanthone, amarolide, acetyl amarolide, 2-dihydroailanthone, ailanthinone, chaparrin, chaparrinone, quassin, neoquassin, shinjulactone, and shinjudilactone [165]. In the mid-1990s, it was confirmed that the reason for the phytotoxic effects of *A. altissima* is ailanthone (Figure 1). Its inhibitory effects on *Brassica juncea*, *Eragrostis tef*, and *Lemna minor* were shown in bioassays [166], and then confirmed on *Lepidium sativum* [165]. Later, De Feo et al. [32] identified several additional quassinoid derivatives and showed that ailanthinone (Figure 2), chaparrine, and ailanthinol B (Figure 2) also have an inhibitory effect on germination and growth of the roots of *Raphanus sativus* L., *Lepidium sativum* L., and *Portulaca oleracea* L. The most powerful allelochemical is ailanthone. Since the inhibitory effect of the extracts depended on the solvent and the plant organ, the authors suggested that the quality and quantity of allelochemicals may vary in different organs of *A. altissima*. Ailanthone also demonstrated anti-bacterial, anti-inflammatory, and anti-tumor activities [167]. Recently, a number of other metabolites has been identified in the cortex of *A. altissima*, among which tetracyclic triterpenoids (altissimanins) and terpenylated coumarin (altissimacoumarin) are of particular interest as potential allelochemicals [168].

The phytotoxicity of ailanthone suggested its use as a natural herbicide. Heisey [169] showed that the phytotoxin was mostly concentrated in bark of roots (up to 1 g/kg DW) and stems of *A. altissima*. In the greenhouse, treatment with root bark extract of ailanthone at a dose equivalent of 0.5 kg/ha resulted in the complete death of five among seven plant species [165]. Studies have shown that the post-emergence herbicidal activity of ailanthone was higher than pre-emergence one [165,166]. Similar results were showed by the field tests. Post-emergence treatment of 17 species of weeds and crops with stem bark extracts led to the death or severe damage of most species in 5–6 days [170]. Ailanthone, apparently, was not autotoxic because neither extracts [35] nor aylanton in the maximum dose of 8 kg/ha [165] did not affect the growth of the *A. altissima* plants, but the mechanism(s) of this phenomenon are unknown. Heisey [165] suggested acylation and glucosylation of ailanthone and subsequent compartmentalization in vacuoles.

The experimental results showed that ailanthone has a powerful herbicidal and post-emergence effect, which can be compared to synthetic herbicides such as glyphosate and paraquat [165]. Its disadvantages include low selectivity and rapid degradation by soil microorganisms. It turned out to be toxic for both weeds and crops, both for monocots and dicots (to a greater extent), but some species from the *Malvaceae* family such as cotton (*Gossypium hirsutum*) and velvet leaf (*Abutilon theophrasti*) proved to be resistant [165]. Due to microbial activity, the ailanthone lost phytotoxicity after five days in normal soil, but kept it high for 21 days in sterile soil [165]. The rapid decomposition of ailanthone was also confirmed in field conditions [170]. Low persistence is beneficial from an environmental point of view, but it requires repeated treatments during the growing season.

**Figure 3:**
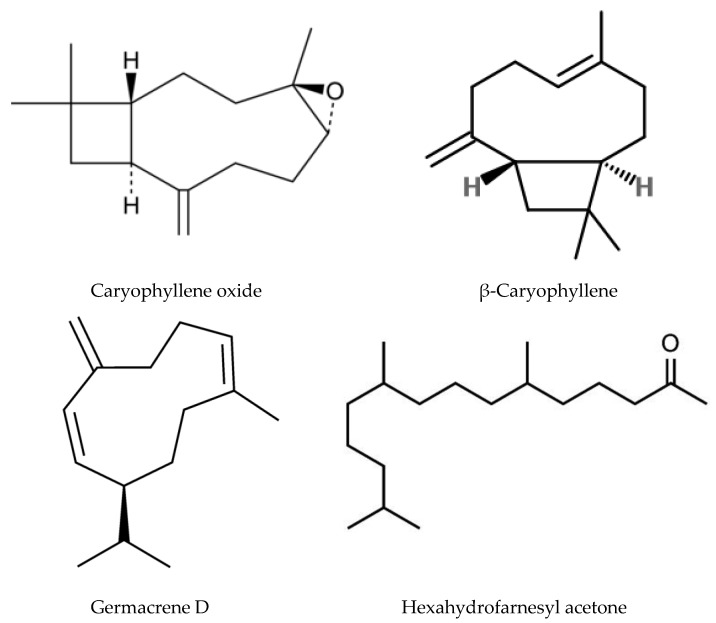
Compounds of the *Ailanthus altissima* essential oil.

Aside from the quassinoids, other compounds of *Ailanthus* may have allelopathic activity. The volatile oil and phenolic constituents of leaves and the phytotoxic properties of extracts were studied by Albouchi et al. [37]. In total, 139 substances were identified in leaves’ essential oils, which were mainly non-terpenic compounds (tetradecanol, heneicosane, tricosane, and docosane) and sesquiterpene hydrocarbons (α-curcumene and α-gurjunene). The composition of the essential oils depended on the part of the plant, the stage of its development and the geographical area where it was collected. Methanol extracts inhibited the germination and growth of the roots of *Daucus carota* L. The phytotoxic effect correlated better with extracts with a higher content of phenols. The reason for that can be such putative allelochemicals as gallic acid, chlorogenic acid, glucosylated quercetin, and glucosylated luteolin [37]. In another study, the composition of essential oils from various organs of *A. altissima*—roots, stems, leaves, flowers, and samaras was evaluated [171]. The essential oils’ content varied from 0.012 (roots) to 0.083% (stems). There were identified 69 compounds, the content of which strongly depended on the plant organ. Although in this and previous work, the assessment was carried out on *Ailanthus* plants from Tunisia, only 17 compounds were the same in both studies and only four in essential oils’ leaves. The reason may be genetic differences, environmental factors, and various metabolic pathways [171]. Essential oils from various organs of *A. altissima* inhibited the germination and growth of *Lactuca sativa*. The observed phytotoxic effect can be associated with caryophyllene oxide, b-caryophyllene, germacrene D, and hexahydrofarnesyl acetone (Figure 3), which prevailed in essential oils from flowers and leaves, stems, roots and fruits, respectively [171].

### 3.2. Juglone

**Figure 4:**
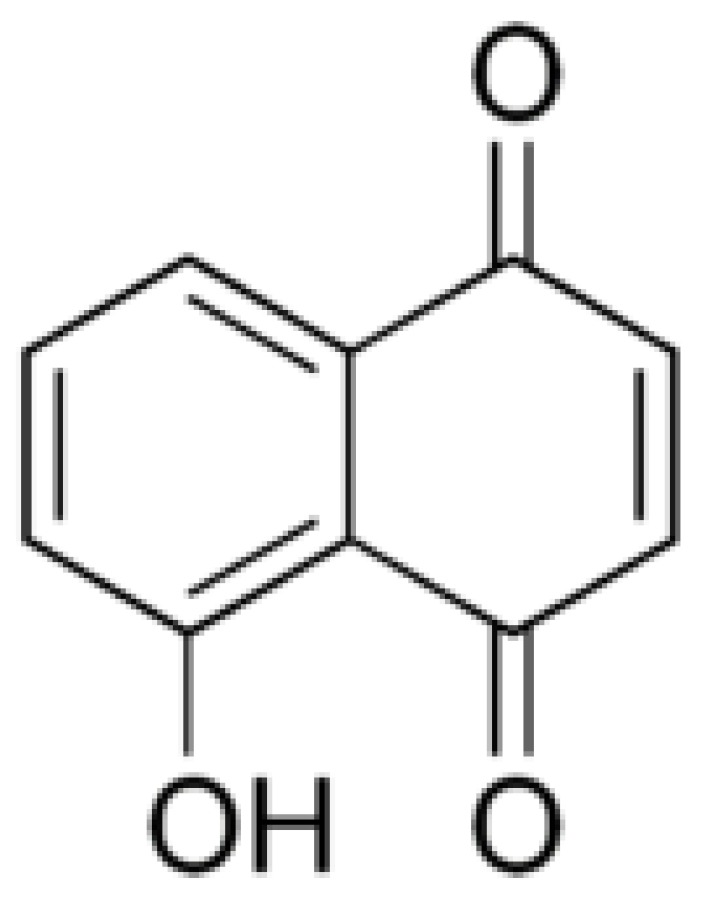
Juglone.

Juglone (5-hydroxy-1,4-naphthalenedione, Figure 4) is the main allelochemical in the *Juglans* genus. Juglone was first extracted in 1856 from a pericarp of walnut [172]); and somewhat later it was synthesized, and its structure was determined [173]. Massey [86] suggested that the toxic for tomato plants compound made of the black walnut extract might be the juglone; and soon Davis [174] demonstrated for the first time that the substance from hulls and roots of black walnut that was toxic for tomato and alfalfa plant was juglone.

Juglone belongs to the naphthoquinones, a group of secondary metabolites with cytotoxic properties that are widespread in Nature. Naphthoquinones are synthesized in more than 200 species of higher plants representing the *Droseraceae, Ebenaceae, Juglandaceae, Plumbaginaceae* and other families [175]. As of today, juglone was extracted from plants of the following species in the walnut family (*Juglandaceae*): Persian walnut (*J. regia* L.), black walnut (*J. nigra* L.), Manchurian walnut (*J. mandshurica* Maxim.), pecan (*Carya illinoensis* (Wangenh.) K. Koch), Caucasian walnut (*Pterocarya fraxinifolia* Lam.), etc. [176]. Studies demonstrated that juglone was able to produce inhibitory effects on the insect larval development, sedative effects on fish and animals; besides, it developed antimicrobial, antifungal, and antiparasitic activity [177]. In addition, juglone manifested activity against many types of tumors [178].

Juglone could be found in various parts of walnut trees: leaves, stems, fruit hulls, inner bark, and roots [88]. According to [179], the maximum concentration of juglone in black walnut trees was noted in fruits, in leaves it fell almost two-fold, in vegetative and flower buds by 5–7%, and in conducting tissues as unit percents and fractions of a percent of the content in fruits. Juglone content in the walnut kemel varied from 7 to 19 mg/100 g, and in the thin skin (pellicle)—from 190 to 727 mg/100 g depending on the variety [180]. While in the fresh walnut leaves the juglone content ranged from 13.1 to 1556.0 mg/100 g of dry weight [181]. Juglone content in annual shoots of four walnut varieties was increasing from the end of May, and reached its maximum in mid-July (average of about 200 mg/100 g DW). After that it was decreasing, and dynamics looked similar for all the varieties [182]. Similar dynamics was demonstrated for walnut leaves: accumulation peak was observed in mid-July (48.2–108.0; average of 73.8 mg/100 g of fresh weight in nine grades) [183].

Unlike other wood allelopathic cultures, which phytotoxicity was firstly examined using extracts and filtrates, and only afterwards the effect of main components was investigated, with the walnut main attention being paid to juglone, the active substance, and not to the vegetable extracts. Juglone inhibited growth of coniferous species seedlings (*Larix leptolepis*, *Picea abies, Pinus strobus*, and *P. sylvestris*) at a concentration of 10^−4^ M and was lethal at concentration from 10^−2^ M to 10^−4^ M depending on the species [184]. In addition, 16 herbaceous and woody species also were sensitive to juglone, but seed germination and radicle elongation were less affected than shoot elongation and dry weight accumulation [85]. Juglone inhibited the algal species growth under laboratory conditions, but its potential for aquatic management purposes is limited, as it appears to be more toxic for fish [185]. At the same time, juglone is one of the very few allelochemicals that is capable of also stimulating action. It stimulated the growth of the *Cucumis melo* roots and shoots, but nothing is known about mechanisms of this stimulation [87].

Numerous studies were conducted to study the juglone phytotoxicity mechanism. Studies performed using hydroponically grown *Zea mays* L. and *Glycine max* L. Merr. Soy plants showed that juglone inhibited the following physiological parameters: photosynthesis, transpiration, stomatal conductance, leaf and root respiration [186], H^+^-ATPase activity, water uptake, and acid efflux [177]. Juglone decreased chlorophyll *a* and *b* contents and reduced some anatomical structures (xylem vessel and bundle radius of stem, stomata length and stomata number of the cotyledons) of the cucumber seedlings [187]. Bohm et al. [188] showed that exposure to juglone led to significant increase in phenylalanine ammonia-lyase activity, lignin content and its *p*-hydroxyphenyl (H) monomer and decrease in soluble and cell wall-bound peroxidase activities in the roots of soybean (*Glycine max* (L.) Merrill).

These works indicated the effect of juglone on various processes in plants, but the main mechanism of its action was participation in oxidative stress. The naphthoquinones’ biological role lies in redox cycling, i.e., a cyclic process of reducing the compound followed by (auto)-oxidation of the reaction product under concomitant generation of reactive oxygen species [189]. The ability of juglone to generate reactive oxygen species was confirmed in many studies; however, cellular, biochemical and transcriptional changes involved in this plant response are of particular interest. Such studies appeared only during the latest decade. The transcriptional activity of glutathione transferase gene encoding important cytoprotective enzyme was significantly enhanced in maize seedlings reacting to the juglone-induced oxidative [190]. Exposure of rice seedlings to juglone induced reactive oxygen species production and calcium accumulation in roots [191]. Large-scale analysis of the transcriptome demonstrated changes in transcript levels of genes related to phytohormone metabolism, cell growth, cell wall formation, chemical detoxification, etc. The data obtained suggest that the inhibition of root elongation was passing via abscisic, jasmonic, and gibberellic acids, and antioxidant enzymes were involved in protection against juglone toxicity. Tobacco seedlings cultivated on nutrient medium supplemented with juglone led to inhibiting the roots growth and increasing the reactive oxygen species content there [192]. Plants reacted to stress by upregulation of two proline synthesis genes and downregulation of a proline catabolism gene increasing the proline concentration, which ensured juglone-induced changes mitigation. Using the *Lactuca sativa* L. seedlings, a complex mechanism of phytotoxic effect of juglone was demonstrated, which inhibited mitosis, changed mitotic phase index, induced creation of reactive oxygen species, and changed levels of reactive nitrogen species [176]. Ultimately, that led to mitochondrial dysfunction, DNA fragmentation, and programmed cell death in root tips. Studies on maize coleoptile segments showed that juglone increased the H_2_O_2_ generation, which was primarily produced in the cytosolic and cell wall area, and the activity of antioxidative enzymes involved in degradation of H_2_O_2_, where catalase was the key element [193]. This was confirmed at the molecular level in Sytykiewicz et al. [194], which stated that treatment of maize and wheat seeds with juglone led to upregulation of *Cat1*, *Cat2*, and *Cat3* genes encoding the catalase isozymes and significant enhancement in CAT activity. A similar effect juglone had on prokaryotic cells, in particular, *Microcystis aeruginosa*, one of the most toxic cyanobacteria. The suppression of cell proliferation at a concentration of 3.0–9.0 mg/l resulted in a noticeable increase in the activity of the antioxidant enzymes SOD and CAT, which indicates the juglone induction of oxidative stress in the *M. aeruginosa* cells [195].

Juglone herbicidal activity was estimated by spraying potted plants including four types of weeds and wheat (*Triticum vulgare* Vill.) and barley (*Hordeum vulgäre* L.) as the control plants [196]. Treatment at a concentration of 5.74 mM had a lethal effect on the *Papaver rhoeas* L. and inhibited seedling elongation and affected fresh weight of the weed species, but not crops. Chlorophyll contents were decreased with all the species, but to a lesser extent in wheat and barley compared to weeds. These differences could indicate a certain selectivity of juglone, since all weed species were dicots. It has been shown that the mature fruits of the Persian walnut (*Juglans regia*) in addition to juglone contain various phenolic acids that have an allelopathic effect (chlorogenic, caffeic, p-coumaric, ferulic, sinapic, ellagic, and syringic acids) [180].

Joint cultivation of not only the nuciferous together with the herbaceous agricultural crops (agroforestry), but the use thereof in mixed plantations of forest species could be of interest. It also requires assessment of the allelopathic wood species compatibility with other trees; but due to the longevity of such plantations, accumulation of allelochemicals in soil should be taken into account, which is less important for the herbaceous crops. Juglone level in soil, and therefore its toxicity to plants, is determined by a balance between its entry through washing out from leaves, tree waste, roots exudation and its movement to the underlying soil layers and decomposition in soil. Juglone is slightly soluble in water, which limits its leaching in soil, but interaction with soil particles accompanied by microbial activity could reduce its concentration. Rietveld [85] reported that walnut allelopathy seemed to be a characteristic feature for poorly drained soils, where chemical and microbial oxidation was restricted, and with well-drained soils it could missing even for sensitive species. Later, bacteria that could degrade juglone were isolated from soil beneath the black walnut trees [197]. Von Kiparski et al. [198] showed that juglone concentration in soil pore water did not exceed the inhibition threshold reported for typical intercrops such as maize and soybeans. In soil, juglone was exposed to both microbial degradation and abiotic transformation reactions. However, the authors reported that in black walnut plants substantial concentrations of juglone were released into the rhizosphere soils, and in acidic soils were low in organic carbon, and fertility juglone could be accumulated up to phytotoxic levels. Strugstad and Despotovski [199] reported on the persistence of juglone in the soil; thus, after removing the walnut trees toxicity could persist for up to one year following removal. On the other hand, it was shown that juglone could be released from Manchurian walnut roots into the soil in a sufficient quantity, but it rapidly degraded due to interactions with soil factors [200]. All this indicates the need for long-term field tests to determine the optimal composition of mixed plantations under specific soil and climatic conditions. Yang et al. [200] demonstrated that survival of Manchurian walnut seedlings was higher in the larch and mixed-species plantations than in the pure Manchurian walnut plantation, and they were growing better in greenhouses in larch and mixed-species soils than in the Manchurian walnut soil. The reason for the Manchurian walnut growth stimulation could lie in increasing the soil microbial populations and the enzyme activities under the larch root exudates influence. Other studies of Manchurian walnut (*Juglans mandshurica*) and larch (*Larix gmelinii*) mixed plantations showed that Manchurian walnut root orders under interspecific competition possess limited plasticity compared to larch [201]. The authors suggested that the Manchurian walnut “strategy” is based on competition via juglone exudation and not exploitative competition, resulting in a lower plasticity in uptake-related root traits.

### 3.3. Mimosine

**Figure 5:**
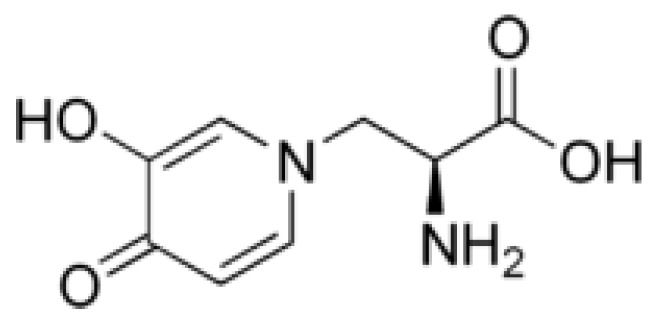
Mimosine.

The main allelochemical in *L. leucocephala* is mimosine [β-(3-hydroxy-4-pyridon-1-yl) -L-alanine] (Figure 5), which was extracted in the 1930s from the leaves of this plant, as well as from sprouts and roots of *Mimosa pudica Benth* [202], and later its chemical structure was determined [203].

Mimosine is a non-protein amino acid structure analogue of dihydroxyphenylalanine with a 3-hydroxy-4-pyridone ring instead of a 3,4-dihydroxyphenyl ring [204]. Mimosine is found in all parts of *L. leucocephala*, but its content can vary by several dozen-fold: the lowest in xylem or developing flowers, the highest in mature seeds [205,206]. The mimosine content in the *Leucaena* leaves ranged from 2.77 to 5.75% of dry weight depending on variety [207]. In another study with similar results amount of mimosine in leaves varied between 3.75 and 5.5% of dry weight depending on the type of leaves, season, and soil quality [208]. It was also shown that the mimosine content changes across the growth stages: as the leaves develop from non-flushed to mature, the mimosine content decreased from 38.8 to 11.1 µmol/g of fresh weight, whereas during seed ripening it increased from 19.7 to 171.3 µmol/g of fresh weight [175].

Intensive studies have shown that mimosine is of interest for medicine and agriculture, as it exhibits anti-cancer, anti-inflammation, anti-fibrosis, anti-influenza, anti-virus, herbicidal and insecticidal activity [202]. Studies on mammalian cells shown that mimosine inhibits DNA synthesis, which prevents the formation of the replication fork by altering deoxyribonucleotide metabolism [209] and is able specifically and reversibly block cell cycle at late G1 phase [210]. In addition, mimosine can chelate divalent metallic ions and 5′-phosphate (PLP), which leads to inactivation of a wide range of enzymes that depend on divalent metal ions or PLP [211]. The phytotoxicity of mimosine was demonstrated in 1966 on seedlings of *Phaseolus aureus* [25]. Subsequently, the inhibitory activity of mimosine (mainly on growth) was also shown on other plant species: *Oryza sativa* [212], *Bidens pilosa* L. *var radiata* Schertt, *Brassica rapa var. amplexicaulis*, *Lolium multiflorum* L., *Phaseolus vulgaris* L. *var. humilis* [205], *Sesbania exaltata*, and *Senna obtusifulia* [213]. The use of mimosine on plants led to a decrease in chlorophyll content [213] and inhibition of activity of various enzymes: antioxidant [212,214] or lignin biosynthesis [214]. It was demonstrated that mimosine when mixed with FeCl3 at a ratio of 4:6 becomes non-toxic to plants—this effect of mimosine inactivation with iron had previously been reported in animals [205]. The strong induction of accumulation of mimosine by acute UV-C exposure suggests that, like several secondary metabolites, mimosine can participate in general oxidative stress modulation, acting as a hydrogen peroxide and superoxide anion quencher [215].

In rare cases, mimosine can be a stimulant. It was shown that mimosine at a concentration of 1 mM inhibited cell proliferation of major phytoplankton groups, but increased the cell number of dinoflagellates [216]. The growth of many dinoflagellate species under laboratory conditions is problematic, and mimosine can greatly enhance the isolation and culture of this group of phytoplankton. The data on autotoxicity of mimosine are ambiguous: it either did not affect the growth of the *L. leucocephala* seedlings [25], or only inhibited the shoot growth [47], or affected the germination of *L. leucocephala* and *Mimosa pudica*, another producer of mimosin, only in high concentration [205]. In soil, mimosine showed high biostability—only 5.30 and 0.16% of mimosine decomposed after one and five days, respectively [205]. Thus, mimosine shows promise for use as a bioherbicide. To our knowledge, herbicidal activity of mimosine has not been evaluated in field, but it has been shown that *Leucaena* can reduce plant productivity in agroforestry systems [79].

**Figure 6:**
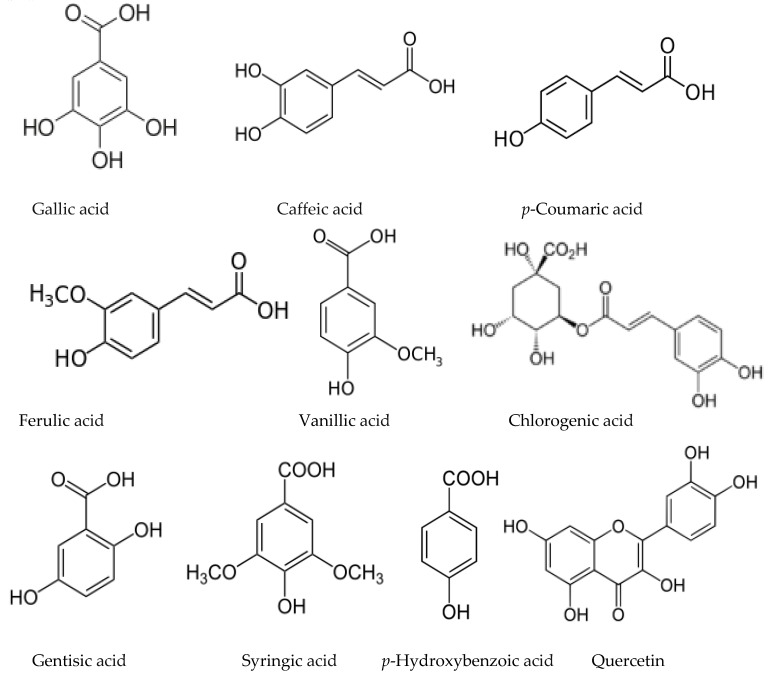
Phenolic allelochemical compounds.

In addition to mimozine several phytotoxic allelochemicals were also identified in Leucaena, such phenolic compounds as quercetin, gallic, protocatechuic, p-hydroxybenzoic, p-hydroxy- phenylacetic, vanillic, ferulic, caffeic, and p-coumaric acids (Figure 6) [207]. Later on, 18 more flavonoids were identified in the leaves [79]. The possible influence of phenolic compounds and flavonoids on the inhibition of plant growth with Leucaena extracts has been reported in several studies [47,82]. Flavonoids are known to be a class of allelochemicals that effectively block mitochondrial functions [217]. Multiple physiological disorders, including mitochondrial one, observed in Eichhornia crassipes leaf disks treated by leachate of L. leucocephala imply the combined effect of several phytotoxic compounds on multiple target sites at multiple cellular levels [83].

### 3.4. Allelochemicals in the Acacia Species

The allelopathic properties of *Acacia dealbata* and other *Acacia* species have long been known, and Reigosa et al. [67] suggested that *p*-hydroxybenzoic, protocatechuic, gentisic, *p*-coumaric, and ferulic acids may be responsible for its toxicity. However, the first study to identify non-volatile allelochemicals has been carried out quite recently. Analysis of the extracts of *A. dealbata* demonstrated that the leaves mainly contain resorcinol (phenol), maculosin (cyclodipeptide), moretenone (triterpene); while stigmasterol (steroid), D-α-tocopherol quinone (quinone), and lupanin (quinolizidine alkaloid) were identified in pods, and methyl *p*-anisate (phenylpropanoid), *p-*anisyl alcohol (phenylpropanoid), stigmasterol, and anisal (benzaldehyde) in flowers [72]. The analysis of phenolic compounds in *A. melanoqlon* showed a predominance of luteolin and apigenin (flavons) in roots, and 4-hydroxy-3-methoxybenzyl alcohol in leaves [74].

The role of volatile compounds (VOCs) of plants from the *Acacia* genus on the environment was first reported by Souza-Alonso et al. [218], who evaluated the impact of VOCs from various parts of *A. dealbata* on plant development and identified them using GC and GC/MS. The composition of the volatile substances of leaves, flowers and litter differed both qualitatively and quantitatively. A total of 67 substances with a predominance of aliphatic compounds, nine of which were common to the three isolates, were detected. VOCs, mainly from flowers, where high levels of heptadecadiene, *n*-nonadecane, octadecene, and *n*-tricosane could be responsible for inhibition, reduced germination and growth of *Trifolium subterraneum*, *Lolium multiflorum*, and *Medicago sativa*. The increase in peroxidase activity and malodialdehyde content was noticed in two species, which suggests oxidative stress and membrane damage [218]. In addition, the VOCs of the *A. dealbata* flowers decreased germination and growth of their own seedlings, whereas throughfall leachate, on the contrary, stimulated the growth of their own roots [69].

**Figure 7:**
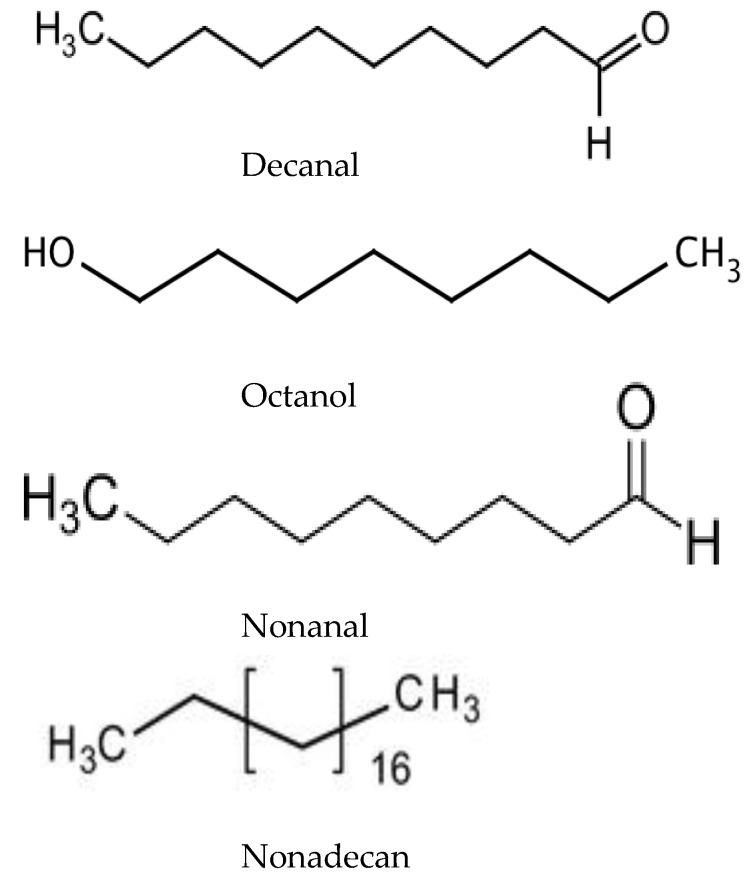
Components of the *Acacia essential* oils.

Later, similar studies with VOCs were carried out on *Acacia cyanophylla* [219] and *Acacia longifolia*, where the dense atmosphere under thick canopies could be associated with reduction in plant richness [220]. In both studies, significant allelopathic activity was shown against a number of plant species. The number of identified substances was lower: 51 in *A. cyanophylla* and 59 in *A. longifolia*. The composition of VOCs from *A. dealbata* was rather similar to *A. longifolia*, while in *A. cyanophylla* it was significantly different from the other two species. Presumably, this is due not only to species-specificity, but also to different methods of extraction. Only single compounds, such as decanal, octanol, nonanal, and nonadecane (Figure 7), were present in all the species. The composition of VOCs varied significantly within various plant parts. Qualitative and quantitative differences between various parts of plants in VOCs composition, but the similar negative effects on seedlings suggest that they are caused by the entire set of VOCs rather than by a single compound [220]. All of these studies were carried out only under laboratory conditions thus field testing is required.

### 3.5. Allelochemicals in the Eucalyptus Species

**Figure 8:**
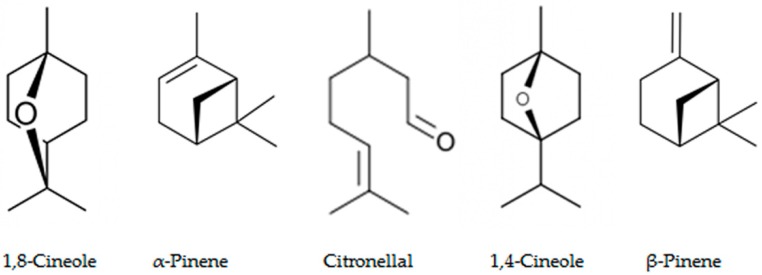
Components of the *Eucalyptus essential* oils.

*Eucalyptus* alleochemicals can be divided into three groups: (1) essential oils (water-insoluble fractions); (2) phenolics (water soluble); and (3) water-soluble volatile fractions. Eucalypts are mainly known for their essential oils, which are widely used in pharmaceutics and cosmetology. These volatile substances regulate transpiration in plants, attract pollinating insects, protect plants from being eaten by animals, and impart resistance to pests and pathogens. Such a high biological activity of essential oils suggests their allelopathic effect. Essential oils are usually derived from the eucalypt foliage by steam distillation (hydrodistillation). They are represented by complex mixtures of monoterpenes (C_10_) and sesquiterpenes (C_15_) with low levels of phenylpropanoids and acyclic hydrocarbon derivatives such as oxides, ethers, alcohols, esters, aldehydes, and ketones [221]. The chemical and biological activity of the eucalypt essential oils is studied fairly well (see reviews [222,223]. The composition of essential oils varies greatly in different eucalypts. In majority, the main components are monoterpenes 1,8-cineole (Figure 8) and α-pinene (Figure 8). The content of 1,8-cineol is especially high in the following species: *E. cinerea*, *E. globulus*, and *E. camaldulensis*, reaching 90%, and such oils are used in medicines, while the content of α-pinene usually does not exceed 20% [223]. Essential oils of *E. citriodora* are rich in citronellal (49.5–87%) (Figure 8) and citronellol (8–20%) and is used in perfumery [222]. The composition of the eucalypt essential oils can vary significantly not only depending on the type and variety, but also on geographic location, climate, soil, season, extraction method, and other factors that should be considered when conducting experiments.

Monoterpenes, the main constituents of essential oils, compose a group of compounds with a diverse range of different functional groups, which implies their different biological activity. Still, there are much fewer studies of individual components of essential oils. In order to evaluate the allelopathic activity of eucalypts either the eucalypt essential oils or their main components, 1,8-cineole, α-pinene, and citronellal, are used in experiments. Essential oils with a predominance of these two components inhibited the germination and growth of the test species *Lactuca sativa* (*Eucalyptus urophylla*, [224], *Amaranthus viridis* weeds (*Eucalyptus tereticornis*, [225], *Lolium hardum* and *Hordeum glaucum* (*E. dundasii*, [226], *Sinapis arvensis* L. and *Phalaris canariensis* L. (*Eucalyptus erythrocorys*, [227]. The allelochemistry of the *E. saligna* essential oils was not selective and influenced both monocots (*Poaceae*) and dicots (*Fabaceae*) [54]. On the contrary, the seedlings growth reaction of three one-year weeds on the *E. globulus* essential oils was species-specific: *Portulaca oleracea* L. did not react, *Lolium multiflorum* Lam. radicle growth was inhibited, while in *Echinochloa crusgalli* (L.) Beau. ragicle growth and hypocotyl growth were observed [228]. Essential oils of *E. citriodora*, where citronellal dominated significantly, inhibited the germination and growth of weed seedlings *Bidens pilosa*, *Amaranthus viridis*, *Rumex nepalensis*, and also *Leucaena leucocephala* tree growth [229]. In the laboratory conditions, the same essential oils inhibited the germination and growth of *Parthenium hysterophorus* [230], *Sinapis arvensis, Sonchus oleraceus, Xanthium strumarium*, and *Avena fatua* weeds [231]. Moreover, when spraying in a greenhouse they caused the plants’ death in 7.5–10 and 3%, respectively. These oils also did not have selectivity properties: they inhibited seed germination and seedling growth both in dicotyledons (crops, such as *Cassia occidentalis* and *Amaranthus viridis* and weeds, *Raphanus sativus*) and monocots (*Echinochloa crus-galli* weed, *Triticum aestivum* and *Zea mays* crops) [232]. However, spraying in the greenhouse showed that the essential oils of *E. citriodora* with a predominance of citronellal was more phytotoxic to *C. occidentalis* (a broad-leaved weed) than to *E. crus-galli* (a grassy weed). These data are consistent with the previous studies, where it was confirmed that citronellal is more effective against broad-leaved weeds than cineole [233]. There are only a few studies on the assessment of the essential oil herbicidal activity in the field. The treatment of emulsions of essential oils from *E. citriodora* at a concentration of 0.5 and 1% showed very little effect on *C. occidentalis* and *E. crus-galli*, but complete killing of *C. occidentalis* and severe injuries in *E. crus-galli* were observed at 7.5 and 10% [232].

The physiological effect of the eucalypt essential oils on the species that were tested was quite similar. Numerous studies have shown that the essential oils effect on plants in both laboratory bioassays [225,229,234] or greenhouse [230,231,234], regardless of individual components content leding to a significant decrease in chlorophyll content and, in some cases, to rapid electrolyte disruption. These results indicated the adverse effect of the eucalypt oils on photosynthetic and energy metabolism of the test plants.

When evaluating individual eucalypt monoterpenes Romagni et al. [235] showed the effectiveness of 1,8-cineole against grassy weeds, and it was more effective against monocot (*Echinochloa crus-galli*) than dicot (*Cassia obtusifolia*). The inhibitory effect of 1,8-cineole was also confirmed on *Solanum elaeagnifolium* Cav., noxious weed in Australia and other countries [236]. a-Pinene inhibited early root growth of weeds *Cassia occidentalis* and *Amaranthus viridis* and cultivated plants *Triticum aestivum, Pisum sativum*, and *Cicer arietinum* [237]. Citronellal significantly inhibited seedling length and seedling weight of two weeds *A. viridis* and *E. crus-galli* and two crops *Triticum aestivum* and *Oryza sativa* under laboratory conditions [238]. Citronellal’s phytotoxic effect has also been shown to be strongly weed-proof (*Ageratum conyzoides* L., *Chenopodium album* L., *Parthenium hysterophorus* L., *Malvastrum coromandelianum* L. Garcke, *Cassia occidentalis* L., and *Phalaris minor* Retz.) [239]. A significant effect on seed emergence and early seedling growth was observed in laboratory bioassay, while spraying of citronellal resulted in disintegration of cuticular wax, distortion of epidermal cells and stomatal closure. The least resistance was shown by *C. album* and *P. hysterophorus*. The mechanism of monoterpenes effect is not fully understood, but it was similar to the action of essential oils: citronellal reduced chlorophyll content and respiration [233], and it suppressed mitosis in root tip cells, and in some cases even caused enucleation [239]. α-Pinene induced oxidative stress through the enhanced generation of reactive oxygen species, which was accompanied by membrane damage. The plants reacted by activation of antioxidant enzymes as a secondary defense mechanism [237].

There are only a few studies where the phytotoxicity of various monoterpenes and their comparison were examined. They showed differences not only between different substances but also among isomers and substances with a similar chemical structure. Among the four monoterpenes, citronellol, citronellal, 1,4-cineole (Figure 8), and linalool, maximum phytotoxicity on the germination, growth and physiology of *Cassia occidentalis* was caused by citronellal—an oxygenated monoterpenoid with an aldehyde group, while 1,4-cineole proved to have minimal phytotoxicity [233]. Comparison of structural isomers and enantioisomers of pinene on *Zea mays* showed that, in general, β-pinene (Figure 8) was more phytotoxic than α-pinene, but there was no clear correlation between the structures, physicochemical properties of these isomers and their biological effects [240]. Despite the similarity of the structure, 1,4- and 1,8-cineole have different modes of action on two weedy plant species: 1,8-cineole severely decreased all stages of mitosis, while 1,4-cineole decreased only the prophase stage [235].

These results suggest that the herbicidal activity of essential oils is not associated with a single major compound, but with the synergistic effects of several compounds. The citronellal content in the juvenile leaves of *Eucalyptus citriodora* was significantly higher than in the adult leaves—77.7% and 48.3%, respectively, but the essential oils from the old leaves were more phytotoxic [238]. The essential oils of the three eucalypt species, *E. salubris*, *E. dundasii* and *E. spathulata* contained mainly 1,8-cineoleum (52.9–65.5%), but their inhibitory effect on germination and growth of *Solanum elaeagnifolium* was higher than that of pure 1,8-cineole [236].

In the research of the eucalypt phytotoxicity the main focus was made on essential oils and monoterpenes, but its allelochemistry is not limited to these substances. In the early 1970s, del Moral and Muller [241] stated that both terpenes and phenolic acids are important phytotoxic agents produced by *Eucalyptus camaldulensis*. Later, the relation between the phenolic composition of the leaf extracts of *Eucalyptus globulus* and their allelopathic properties was shown by Souto et al. [242]. The leachates of bark, fresh leaves and leaf litter of *E. tereticornis*. *E. camaldulensis, E. polycarpa*, and *E. microtheca* showed the presence of coumaric, gallic, gentisic, hydroxybenzoic, syringic and vanillic acids, and catechol [243]. Bioassay with leachates reduced the germination and the seedling length of *Phaseolus mungo* L., while phenolics may have an inhibitory, neutral or occasionally stimulating effect depending on the substance and concentration. The research of phenolics was increased in recent years. For the aqueous extract of the *E. saligna* leaf litter, a correlation between the phenol content and phytotoxicity on grassland species was shown [54]. Phenolic compounds in leaf extracts of *E. camaldulensis* inhibited the growth and a number of plants physiological processes in laboratory and field studies, and the weeds reacted more strongly than crops [244]. Monoterpenes and phenolic compounds have a different effect on the plants. Del Moral and Muller [241] reported that terpenes influence the annual grassland flora only after becoming adsorbed to soil particles, while the rains leach phenolics from litter into the soil. It was also suggested in [54] that phenolic derivatives may be leached by rainfall, but the phytotoxicity of monoterpenes of *E. saligna* is determined by volatilization from leaf litter.

It is known that the metabolic cost of the terpenoid biosynthesis due to their high level of chemical reduction is significantly higher than that of phenolics (3.11 and 2.11 g glucose/g metabolite), and, in addition, terpenoids are generally sequestered in complex secretory structures to avoid autotoxicity [245]). Based on these calculations, Goodger et al. [246] are hypothesized that phenolics as the cheapest defence metabolites would predominate in the resource-limited seedlings, where as the most expensive terpenoids—in mature trees, once the costs of biosynthesis and foregone photosynthesis could be overcome by enhanced resource acquisition. They confirmed their hypothesis in studies on *Eucalyptus froggattii*, where they demonstrated that the ratio of total phenolics to total terpenoids significantly decreased in an exponential manner with plant ontogeny [246]. It is possible that the same situation is observed in other plant species containing phenolic and terpene allelochemicals. This once again confirms the importance of a long-term assessment of allelopathic effects for woody plants, since they largely depend on various environmental factors that may change significantly during the life of trees.

At the same time, the soil can significantly affect allelopathy: toxins can either accumulate or oxidize and lose their activity depending on the soil composition. It was shown that the inhibitory effect of the *Eucalyptus grandis* × *E. urophylla* leaf leachates on *Brassica shinensis* was more pronounced in the sterilized soils [247]. Authors suggested that soil microbes can alleviate the allelopathic potential of *Eucalyptus*. Biotests demonstated that soil might neutralize or dilute allelopathic agents with the increase of the *E. grandis* plantation age [51]. Much rarer is the conversion of a plant allelochemical into a more phytotoxic compound by soil microbes. Wang et al. [125] found that the soil microbiome biotransformed an allelochemical (-)-epicatechin (EC_50_ 9.2–10.8 mM), released from *Rhododendron formosanum*, into a more phytotoxic active compound, protocatechuic acid (EC_50_ 4.3–4.4 mM). This study revealed the significance of the allelopathic interactions between plants and microorganisms in the rhizosphere.

Except for essential oils (water insoluble fractions) and phenolics (water soluble), another group of eucalypt allelochemicals is widely known and called water soluble volatile fractions. They have been studied relatively recently. Such fractions obtained during steam distillation of *Eucalyptus dundasii* leaves demonstrated phytotoxicity against *Lolium rigidum* Gaudin and *Hordeum glaucum* Steud. [226]. The analysis of their chemical composition was not performed. Later, the compositions of the aqueous volatile fractions for four types of eucalypts were determined by gas chromatograph—mass spectrometry (GC-MS). 1,8-Cineole dominated (37.1–80.1%) in the compositions, while the other major components were isopentyl isovalerate, isomenthol, pinocarvone, *trans-*pinocarveol, α-terpineol, and globulol, depending on the species [248]. These fractions also showed strong phytotoxicity against *Solanum elaeagnifolium* Cav. The analysis of water soluble volatile fractions from other four eucalypt species also proved that they contained mostly 1,8-cineole (about 90%) [249].

The composition of volatile organic compounds in solutions obtained by a natural way was significantly different from solutions obtained by a steam distillation. In total, 28 potential allelochemicals were found in aqueous extracts of the roots of *E. grandis*, and 38 were found in rhizospheric soil extracts, but 1,8-cineole was not found in them [51]. Twenty components, including alkane, aromatic ester, arene, and phenol, were found in both roots and the rhizosphere soils. This fact suggests that a significant proportion of allelochemicals in the soil can be released from the roots. These results are not consistent with data obtained in the research on volatile organic compounds (VOCs) released into water extracts during root exudation, foliage and leaf litter leaching, and leaf litter decomposition in the laboratory or from field soil around *Eucalyptus urophylla* [250]. The studies have proved that VOCs were not extracted from the root exudates in soil water, while 12 VOCs were identified in foliage, leaf litter leachates, and the leaf litter decomposition extracts, nine of which were identical, but differed in concentrations. Terpinen-4-ol prevailed in the compositions of both types of extracts, while 1,8-cineole was 19.8 in foliage and leaf litter, but 8.0% in the decomposited leaf litter extracts. These extracts significantly inhibited seed germination and seedling growth of *Lolium multiflorum* Lam. and *Bidens pilosa* [250].

**Figure 9:**
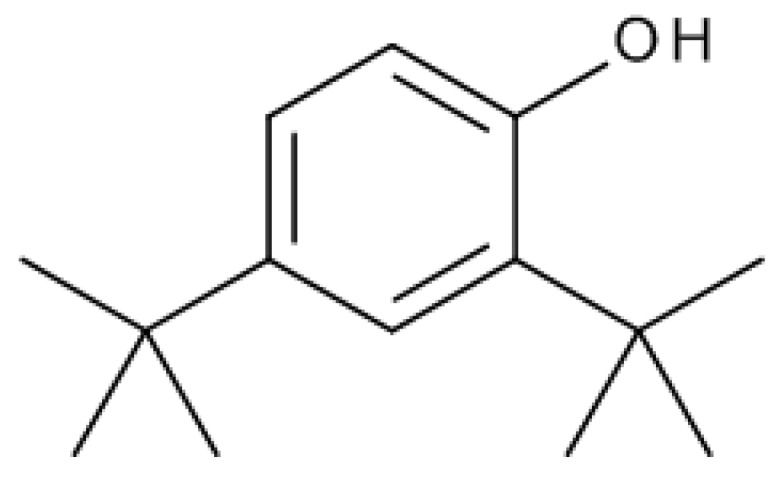
2,4-Di-*tert*-butyl phenol.

The volatile compounds *n*-octane, 2,4-di-*tert*-butyl phenol (Figure 9), and 2,2′-methylene bis (6-*tert*-butyl-4-methylphenol) were the most abundant in the soil of the *Eucalyptus grandis* plantation [51] and were tested on three most common species *Vigna radiata, Raphanus sativus*, and *Lactuca sativa* [251]. These substances were inhibitory for the test plants at high concentrations (2,4-di-*tert*-butyl phenol was the strongest) and stimulatory at low concentrations. The effects on the root activities were more obvious. This fact can explain the poorer vegetation in the eucalypt plantations. This observation was confirmed in [252], where effects of litter leachates of *E. globulus* on root growth of seedlings of 21 species, including grasses, forbs, and trees were compared (*Acacia, Eucalyptus*, and *Dalbergia* species): 15 native species from the USA, Chile or India (the non-native ranges of *Eucalyptus*), and six species native to Australia, in a greenhouse conditions. The root growth of all non-native range species was highly suppressed (45–100%, in average 71%) by the *E. globulus* litter leachates, whereas the effect of litter leachate varied from stimulation to suppression for six species native to Australia (reduction in average by 1%). In the field conditions, the reduction of species composition, richness and height of plant communities under *E. globulus* trees, where the reduction was much greater in the non-native ranges (India, Chile, the USA, Portugal) than in native Australia, was also proved [252].

### 3.6. Allelochemicals in Other Deciduous Species

Woody plants may contain allelochemicals of different chemical nature. From leaf and litter of *Alstonia scholaris* Wang et al. [93] isolated three allelochemicals that were identified as pentacyclic triterpenoids: oleanolic acid, betulinic acid, and ursolic acid. These compounds inhibited the seed germination and radicle growth of *Bidens pilosa* and *Lactuca sativa*, and field studies demonstrated that concentration of ursolic acid in the *A. scholaris* soil was significantly negatively correlated with weed coverage during the summer and winter. In this work, it was demonstrated for the first time that triterpenoids have an inhibitory effect on photosystem II [93]. Another example is related to the quinolizidine alkaloids in legumes. For nitrogen-fixing plants in the Fabaceae family, the nitrogen is not a limiting element, and they differ from other species by the ability to accumulate nitrogen-containing allelochemicals—alkaloids (*Lupinus* spp.) or non-protein amino acid mimosine (*Leucaena leucocephala*). The quinolizidine alkaloids protect plants of the genus Lupinus from pests [253], and it has long been known that they have allelopathic activity [254,255]. The *Lupinus* species are usually herbs or low shrubs, but there is an exception—an arboreous lupine *Lupinus jaimehintoniana*. This 5 to 8 m tall subdominant tree was discovered in south Mexico in the middle of the 1990′s [256]. Villa-Ruano et al. [113] indentified for the first time five quinolizidine alkaloids in leaves, seeds, shoots and phloem of *L. jaimehintoniana*, including lupanine (the most abundant), 5,6-dehydrolupanine, d-thermopsine, sparteine, and nuttalline (traces) and demonstrated the inhibiting effect of the semi-purified alkaloid extracts on the germination of *Lactuca sativa* seeds.

Finally, the limonoid 5α,6β,8α,12α-tetrahydro-28-norisotoonafolin from the Australian tree *Toona ciliata* (Meliaceae) is an interesting allelochemical. Its phytotoxicity was tested by Nebo et al. [100], which showed its inhibitory effect on the seed germination and seedling growth of four standard target species with higher bioactivity levels than the commercial herbicide Logran.

### 3.7. Allelochemicals in Conifers

**Figure 10:**
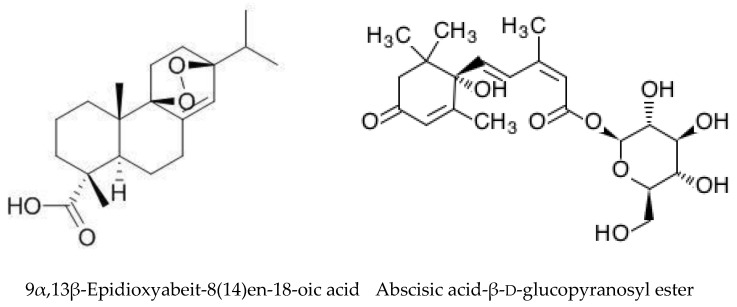
Allelochemicals of *Pinus densiflora*.

**Figure 11:**
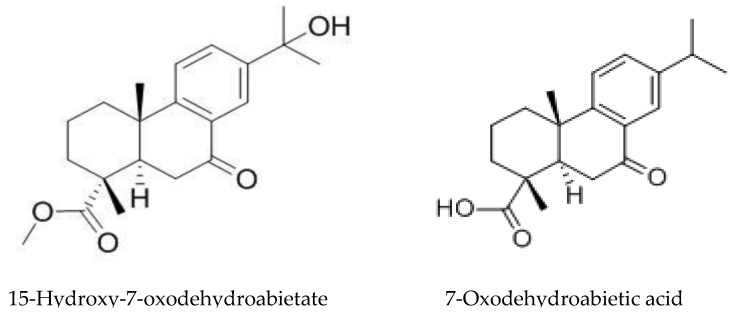
Abietane type diterpenids from *Pinus densiflora.*

Japanese red pine (*Pinus densiflora* Sieb. Et Zucc.) could be found in Japan, China and Korea and is characterized by sparse herbaceous vegetation compared with other forests that suggests the presence of allelopathic effects [257]. Aqueous methanol extract of the *P. densiflora* needles inhibited root and shoot growth of seven plant species, including weeds *Digitaria sanguinalis* and *Echinochloa crus-galli*. Anabietane diterpenoid, 9α,13β-epidioxyabeit-8(14)-en-18-oic acid (Figure 10) was isolated from the extract, and it inhibited root and shoot growth of weed seedlings and could play an important role in the allelopathy of red pine [257]. Abscisic acid-β-D-glucopyranosyl ester (Figure 10) was later isolated from the *P. densiflora* needles, which in bioassays was inhibiting the growth of the *Lepidium sativum* and *E. crusgalli* seedlings at concentration significantly lower than its concentration in soil water of the pine forest, i.e., 0.1 and 2.5 μM, respectively [146]. Another paper demonstrated that aqueous methanol extracts of red pine soil also inhibited the root and shoot growth of six test species, but two other growth inhibitory substances, i.e., abietane type diterpenids, 15-hydroxy-7-oxodehydroabietate and 7-oxodehydroabietic acid were identified there (Figure 11) [147]. These substances inhibited growth of *L. sativum* L. and *Lolium multiflorum* Lam. in aqueous solutions, and also growth of *L. multiflorum* Lam. when added to soil in natural concentrations under litter layer of red pine forest floor. Apparently, these substances are the products of degradation of resin acids that got into the soil under the pine trees through resin and defoliation by soil microorganisms [147]. Two allelochemicals from red pine needles [146,257] were not found in the soil as main inhibitory substances, and their contributions in growth inhibitory activity of the red pine soil could be much less than abietic acid derivatives [147].

*Pinus halepensis* Miller is another relatively thoroughly studied allelopathic pine tree species. This species is distinguished for its ability to rapidly expand from forest plantations into surrounding natural vegetation in Mediterranean regions, which leads to creating monospecific woodlands with decreased biodiversity. Germination and growth of various plant species was inhibited by the *P. halepensis* extracts [148,150], and the release of potential allelochemicals (phenolic compounds) in leaf leachates or root exudates could influence secondary succession [150]. The autotoxicity was also detected for *P. halepensis* [153]. Fernandez et al. [152] showed that the *P. halepensis* allelochemicals composition differed depending on the plants age and tissue: needle leachates were composed mainly of oxygenated terpenoids, whereas roots mainly contained fatty acids. Young plant needles had the highest content of monoterpenes, suggesting their allelopathic role in facilitating the establishment of young pine stands, while high concentrations of caffeic acid in both young needles and old roots could play a key role in giving *P. halepensis* a competitive advantage [152]. Apparently, it was these differences that caused different levels of autotoxicity discovered by Fernandez et al. [153]. Differences in the content of terpene and phenolic allelochemicals depending on age were also shown on eucalyptus [246]. Effect of aqueous extracts from shoots of young *P. halepensis* on germination and growth of 12 target species naturally present in fallow was specific for a species; and the microbial community present in natural soil reduced the toxicity of allelochemicals compared to the sterile soil [153]. Aqueous extracts of young pine needles comprised nearly 50 compounds and mixtures predominantly consisted of phenolics, including those known phytotoxins such as gallic, 4-hydrobenzoic, *p*-coumaric and caffeic acids, and fatty acids. Study of 12 species in the field demonstrated that their abundance decreased along the secondary succession with land closure due to pine colonization; and it should be mentioned that the abundance of four species decreased once pines were present [153].

Allelopathic effects were studied also with other species of pines. Extracts of *Pinus roxburghii* demonstrated inhibitory effect against *Bidens pilosa* in bioassays and greenhouse [155], as well as against five weed species and *Triticum aestivum* using sandwich method [258], but potential allelochemicals were not reported. Essential oils of *Pinus pinea* Linn., where limonene (54.1%), α-pinene (7.7%), and β-pinene (3.4%) were prevailing, inhibited seed germination and seedling growth of weeds *Sinapis arvensis* L., *Lolium rigidum* Gaud., and *Raphanus raphanistrum* L., and at the 2 mL/l concentration was more efficient than herbicide 2,4-D isooctyl ester with the same concentration [154]. Furthermore, recently it was shown that water extracts from the needle litter of *Pinus thunbergii*, *Pinus tabuliformis*, and *Pinus koraiensis*, the main afforestation species in northern China, demonstrated autotoxicity of varying intensity level [156].

**Figure 12:**
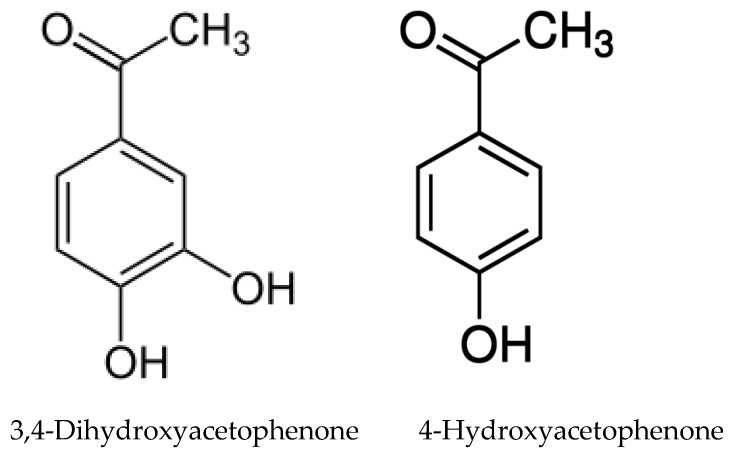
Allelochemicals of *Picea* species.

Autotoxicity could present a problem with other conifers. Schrenk spruce (*Picea schrenkiana* Fisch. Et Mey.) is the native species in Middle Asia and the mountains of Asia, where usually it creates pure forests. This species is of the utmost importance in the mountain ecosystems, because it plays an important role in water and soil conservation, but the natural regeneration of *P. schrenkiana* has been problematic [145]. One of the possible reasons could be found in autotoxic secondary metabolites that are transferred to the soil with litter and root exudates and impede the growth of seedlings of *P. schrenkiana*. Li et al. [4] demonstrated the autotoxicity of litter extracts from Schrenk spruce on seed germination and seedling growth of the same species. From the litter 17 compounds were isolated, including 10 phenolic acids (4-vinylphenol, *p*-hydroxybenzoic acids, 2-hydroxyphenylacetic acid, vanillic acid, gallic acid, gentisic acid, 4-hydroxyphenylacetic acid, β-resorcylic acid, *p*-coumaric acid), and ethyl hematommate. This study was carried out only under laboratory conditions, and tests under natural settings are needed to confirm the role of identified phenolic acids [4]. From water extract of the *P. schrenkiana* needles, the phenolic compound 3,4-dihydroxyacetophenone (DHAP; Figure 12) was isolated, which significantly inhibited growth of *P. schrenkiana* and the six agricultural crops under laboratory conditions [144]. DHAP concentration seemed to be rather high in mature forest soil (0.51 mg/g dry soil) to inhibit the seed germination and seedling growth of *P. schrenkiana* and other co-occurring species. A similar composition, 4-hydroxyacetophenone (Figure 12), was isolated in throughfall, in water extracts of litter and organic soil layer under *Picea abies* and produced an inhibiting effect on root length of spruce seedling [259]. Taking into consideration the great ecological importance of *P. schrenkiana*, the following studies were focused on evaluating the autotoxicity alterations caused by global warming. Ruan et al. [145] proved that with the rising temperatures the DHAP effect on seed germination of *P. schrenkiana* was changing from stimulation to inhibition depending on the concentration. Physiological mechanism of autotoxicity evaluation showed that the moderate concentration of DHAP increased antioxidant enzymes activities in order to protect against reactive oxygen species; but with high concentrations, the activity thereof was decreasing and high concentrations of reactive oxygen species could inhibit the *P. schrenkiana* seedlings growth [260]. This investigation revealed that autotoxicity of P. schrenkiana was affected by the climate warming.

**Figure 13:**
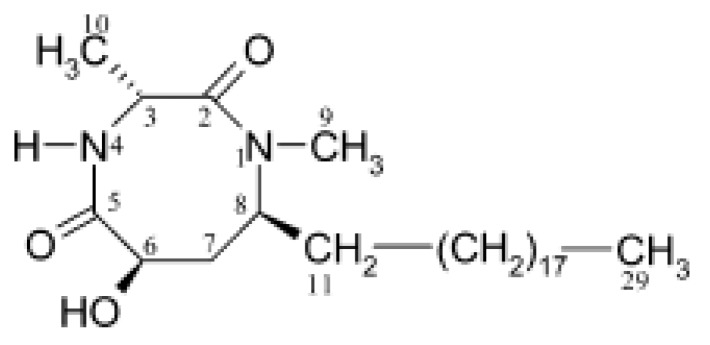
6-Hydroxy-1,3-dimethyl-8-nonadecyl-[1,4]-diazocane-2,5-diketone [107].

Another conifer tree, the Chinese fir (*Cunninghamia lanceolata* (Lamb.) Hook.) has great ecological and economic importance being one of the most important plantation species in Southern China for industrial wood production. However, establishment and productivity decline of the replanted Chinese fir plantations has remained a significant problem [261]. Phenolics were considered as allelochemicals responsible for autotoxicity in the replanted Chinese fir stands. Huang et al. [136] showed that both total phenolic content and water soluble phenolic content in the Chinese fir stump-roots were negatively correlating with the growth of the Chinese fir seedlings. However, later it was proven that individual phenolics and triterpenoid friedelin isolated from toxic Chinese fir soil stimulated the growth of Chinese fir, whereas novel cyclic dipeptide (6-hydroxy-1,3-dimethyl-8-nonadecyl-[1,4]-diazocane-2,5-diketone) (Figure 13) significantly inhibited the growth of Chinese fir [139]. The observation that cyclic dipeptide is a highly active allelochemical was proved in [137], where differences in the cyclic dipeptide contents in the leaf and root extracts, and in the rhizosphere soil from Chinese fir plantations of different age also explained the observed allelopathic effects. However, poor natural regeneration in coniferous forests could be caused also by other reasons. In order to estimate the leaf litter effect on germination and growth of Chinese fir seedlings the artificial plastic litter that has only the physical properties of litter was compared with natural litter that has chemical effects as nutrients and allelochemicals [140]. The results showed that effects of plastic and natural litters did not differ, suggesting that the Chinese fir litters were primarily of physical rather than biological or chemical effect. Job da Silva et al. [62] also showed that the *E. saligna* leaf litter physically suppresses the establishment of grassland vegetation. Chen and Wang [137] reported that allelochemicals of Chinese fir were released into the soil through the roots. It’s quite possible that the Chinese fir allelopathic effects were primarily connected with root exudates rather than litter leachates. In addition, the study was conducted in the controlled environment, and the results in the field could be quite different.

In also should be mentioned that only in the field it is possible to evaluate allelopathic interactions between different types of trees in mixed plantations. Comparison of the Chinese fir monocultures with mixed-species stands containing both the Chinese fir and a broadleaf, non-N fixing species, *Michelia macclurei* showed enhanced growth of Chinese fir [262]. The authors observed reduction of autotoxicity through reduced release cyclic dipeptide from the Chinese fir roots and increased its degradation in the soil due to changes in composition of the soil microbial community. Similar positive effects were also presented in [170], where in the Manchurian walnut plantations mixed with larch the growth of the autotoxic Manchurian walnut was improved. These field studies confirmed earlier greenhouse ones, where water extracts from root, bark, branch and leaf of *Larix gmelini* with low and moderate concentrations accelerated the growth of one-year-old *Juglans mandsburica* seedlings [143].

**Figure 14:**
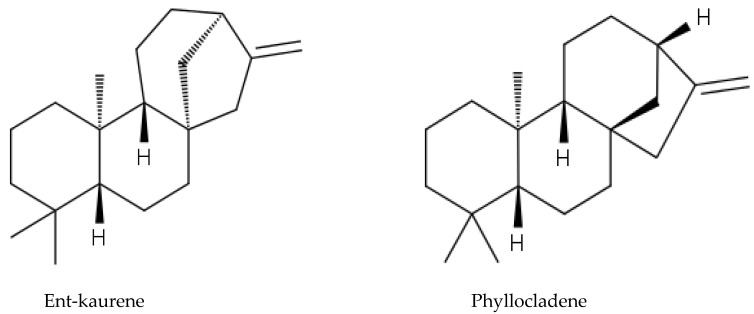
Allelochemicals of *Araucaria angustifolia.*

**Figure 15:**
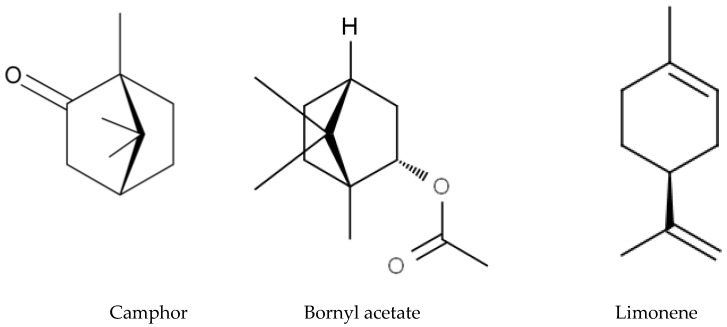
Monoterpens of *Juniperus ashei.*

Allelopathic properties are known also for other conifer species. Needle extracts of *Araucaria angustifolia* inhibit germination and seedling growth of *Lactuca sativa* at high concentrations [135]. The potential allelochemicals were identified as diterpenoids ent-kaurene and phyllocladene (Figure 14), and no phenolic compounds commonly associated with the allelopathic effect were detected in the extracts. The leaf extract of the Wollemi pine (*Wollemia nobilis*), another tree belonging to the *Araucariaceae* family, inhibited the growth of *Lolium rigidum* and wild radish (*Raphanus raphanistrum*) in laboratory bioassays and growth of *L. rigidum* in soil trials [160]. The main constituents identified in the most phytotoxic fraction were terpenes and phenolics. Inhibiting effect of leaf and litter of *Juniperus ashei* Buchh. on the germination of *Bouteloua curtipendula* (Michx.) Torr. was demonstrated using the “sandwich agar method” [142]. In addition, aboveground dry mass of *B. curtipendula* was nearly four times higher than in an intercanopy area compared to the dry mass in the understory and dripline (edge of the tree) in the field experiment, suggesting some negative influence by *J. ashei*. Leaf and litter leachate and volatiles from leaf tissue contained monoterpenes camphor (the most abundant), bornyl acetate, and limonene (Figure 15), which are potentially allelochemicals inhibiting *B. curtipendula* [142]. Allelopathic activity was found also in one of the oldest living tree species, *Ginkgo biloba*. A leaf extract inhibited the growth of four plant species, and an active substance was isolated and identified as the novel compound 2-hydroxy-6-(10-hydroxypentadec-11-enyl)benzoic acid [141].

Studies conducted in the recent years indicated also allelopathic properties of yew (*Taxus baccata* L.). Inhibiting effect of aqueous extracts from arils, leaves and bark on germination and growth of *Raphanus sativus L.* and *Cucumis sativus L.* depended on a type of tissue and concentration and could be associated with the high contents of phenolic in the extracts [158]. It was demonstrated in the ex situ pot experiments that addition of yew needles negatively affected seedling growth, but not seed germination suggesting that the absence of regeneration beneath mature yew canopies may at least be partly related to autotoxicity [157]. Finally, allelopathic properties of terpenoid resin vesicles in the seed coat of fir (*Abies*), hemlock (*Tsuga*), and cedar (*Thuja*) species were demonstrated. Germination of *Arabidopsis* seeds was inhibited with resin vesicle extracts, and damage of resin vesicle prior to stratification had negative effects on their own germination for most species [159].

Unlike deciduous, in conifers, the mechanisms of action of allelochemicals are poorly understood. In addition to changes in the activity of antioxidant enzymes under the action of DHAP [260], the inhibitory effect of needle extracts of various pine species [148,156] and *Taxus baccata* [158] on the content of chlorophyll and photosystem II was also shown. The synergistic effect of the conifer allelochemicals is also noteworthy. The addition of two allelochemicals from the soil of *Pinus densiflora* separately increased the shoot and root growth of *L. multiflorum* by 49.7–57.6% and 34.2–39.7%, respectively, compared with the control, but their mixtures increased the growth only by 11.5–15.7% [147]. Synergism was also observed for other phytotoxins. Blum [263] noticed that mixtures of phenolic acids and other organic compounds can cause inhibitory effects even though concentrations of individual compounds were well below inhibitory levels. Wang et al. [125] found the synergistic effect of the *Rhododendron formosanum* allelochemicals: 10 μg/g soil of (-)-catechin combined with 10 μg/g soil of protocatechuic acid caused the inhibition of the *Lactuca sativa* seed germination by 36.7%, while applied separately 750 μg/g soil of (-)-catechin inhibited germination only by 13.4%, and 10 μg/g soil of (-)-catechin had no effect at all.

## 4. Biosynthesis of Allelochemicals

### 4.1. Juglone Biosynthesis

Many toxic secondary metabolites are stored in the plants producing them in an inactive form, and juglone is no exception. In 1950, Daglish [264] showed that walnut nuts contain juglone as glycoside of 1,4,5-trihydroxynaphtalene and not as α-hydrojuglone, as previously suggested. Later on, various species from the *Juglandaceae* family showed that juglone accumulates in their tissues in its glycosylated form, hydrojuglone glucoside (HJG; 1,5-dihydroxy-4-naphthalenyl-β-D-gluco- pyranoside) to reduce its autotoxicity [265]. The release of juglone from glycoside occurs in plants in two stages: first, β-glucosidase catalyzes hydrolysis to hydrojuglone, which then is chemically oxidized to form a toxic juglone [199].

In nature, 1,4-naphthoquinones can be synthesized in several ways, but juglone is synthesized in the *o*-succinylbenzoate metabolic pathway, which consists of seven reactions leading to the formation of 1,4-dihydroxy-2-naphthoate (DHNA), from which phylloquinone is then synthesized in plants and menaquinones in bacteria [265]. Müller and Leistner [266] showed that 1,4-naphthoquinone is involved in the biosynthesis of juglone in walnut and, apparently, the synthesis of juglone is carried out in two stages: first, decarboxylation of DHNA to 1,4-naphthoquinone, which then turns into juglone by hydroxylase [265]. This assumption was confirmed by McCoy et al. [267] who used the targeted metabolic profiling and comparative RNA sequencing (RNA-seq) to study expression of the phylloquinone pathway in various organs of black walnut. It has been shown that the DHNA biosynthesis genes are expressed in roots to support production of metabolites other than phylloquinone. These results indicate that juglone is *de novo* synthesized in black walnut roots from DHNA derived via the phylloquinone pathway [267].

A number of plant species are resistant to juglon, but the molecular mechanisms of this resistance are not studied at all. It is unclear whether they have enzymes that facilitate detoxification and/or proteins that regulate juglone exclusion, transport, and/or sequestration/ compartmentalization [265].

### 4.2. Mimosine Biosynthesis

Mimosine is synthesized from 3,4-dihydroxypyridine and O-acetylserine by the mimosine synthase (EC 2.5.1.52), which is the isoform of cysteine synthase (O-acetylserine (thiol) lyase, OAS-TL), and the pyridine ring of mimosine appeared to be derived from lysine [206]. Biosynthesis of mimosine (β-substituted alanine) represents an important branching point between primary metabolism (cysteine biosynthesis for protein assembly) and secondary metabolism (mimosine accumulation) [268]. The biosynthesis of these substances is identical to the formation of O-acetylserine, which is then catalyzed by the OAS-TL isoforms into cysteine or mimosine in the presence of sulfide or 3,4-dihydroxypyridine, respectively [268]. The first attempt to isolate the mimosine biosynthesis gene was made in 2014, when Yafuso et al. [269] isolated the OAS-TL gene from *Leucaena*, expressed it in *E. coli*, and evaluated the possibility of a recombinant enzyme to synthesize mimosine and cysteine. However, it turned out that cytosolic OAS-TL is specific for only cysteine synthesis and does not catalyze mimosine formation. The *Mimosa pudica* Mill enzyme has similar properties: the cloned cytosolic OASTL 1275 bp long cDNA expressed in *E. coli*, but enzyme produced cystein only [270]. Harun-Ur-Rashid et al. [271] was the first to obtain the enzyme involved in the synthesis of mimosine. They cloned the 1275 bp long cDNA for cytosolic Cy-OASTL from *Leucaena leucocephala*, and the resulting enzyme showed a dual function of cysteine and mimosine synthesis. Since the apparent *kcat* for Cys production is more than six times higher than for the synthesis of mimosin, and the apparent *Km* is 3.7 times lower, it is likely that for this enzyme synthesis Cys is the preferred route [271].

Mimosine accumulates in plants in large quantities, which requires much energy, as well as deposition of carbon and nitrogen resources. Negi et al. [211] estimated that if it were not for the synthesis of mimosine, the growth of *L. leucocephala* plants would have increased by at least 20%. They suggested that mimosine acts as a source of carbon and nitrogen under stressful conditions, when the availability of nutrients becomes limited, and this explains the resistance of *L. leucocephala* to drought. Mimosine catabolites can be used as nutrients in root nodules, thereby playing a role in the leucocephala tree [206]. Regulation of mimosine accumulation by environmental factors also suggests its role as a nutritional reserve source [268]. Enzymes were isolated from the *L. leucocephala* tissues that decompose mimosine to 3,4-dihydroxypyridine (3,4DHP), pyruvic acid, and ammonia (by CN lyase [25]) or to 3-hydroxy-4-pyridone (by mimosinase [272]). The herbicidal activity of 3,4 DHP was first shown by Xuan et al. [273] on the growth of seedlings of *Echinochloa crus-galli*, and it was about four times lower than that of mimosin. Smith and Fowden [25] suggested that the lack of effect of mimosine and dihydroxypyridine on growth of the *Leucaena* seedlings might be due to the ability of *Leucaena* to further metabolize dihyroxypyridine. This is evident from the absence of dihydroxypridine in the *Leucaena* seedling extracts. The gene encoding the mimosine-degrading enzyme was recently isolated from *Leucaena leucocephala* and described as C-N lyase [211]. The authors of this study suggested the compartmentization model according to which mimosine is synthesized and stored in the cytoplasm, while mimosinase is localized in the chloroplast, and the substrate becomes available for the enzyme under stress conditions (e.g., drought), which may cause damage to the chloroplast membrane. Further studies are needed to identify the dihydroxypridine degradation gene.

The metabolic pathway of mimosine has not been studied enough yet, and current data are not consistent. Mimosine was shown to be present at all stages of the early development of the *L. leucocephala* seedlings, and its accumulation changed both with time and under influence of such factors as light, mechanical damage, salicylic acid, and auxins [268]. On the other hand, Rodrigues-Corrêa et al. [215] also studied the effects of stress factors on seedlings of *L. leucocephala* spp. *glabrata* and found that salicylic acid had no effect, while jasmonic acid, an ethylene-releasing compound, ethephon, and UV-C radiation increased mimosine levels in roots and shoots. At the same time, application of four jasmonate elicitors that mimic the herbivores and wounding stresses to the *L. leucocephala* seedlings caused no change in the content mimosine in leaves, but increased the accumulation of 3,4-dihydroxypyridine, the product of the mimosine degradation, under jasmonoyl-l-isoleucine elicitation [274]. This showed that mimosine belongs to the constitutive metabolite, while 3,4-dihydroxypyridine belongs to the inducible metabolite, and jasmonoyl-L-isoleucine elicitation might activate the degradation of mimosine into 3,4-dihydroxypyridine [274].

A thorough understanding of the mimosine metabolism pathway and its regulation under the influence of endogenous and external factors are fundamental to its metabolic engineering in order to create transgenic *L. leucocephala* plants. The high productivity and protein content of the *Leucaena* foliage can help to solve the problem of insufficient animal feed in developing countries, but this opportunity is limited by mimosine toxicity. Reduction of toxicity is possible in two ways—either by blocking biosynthesis or increasing the degradation of mimosin, and here the enzyme mimosinase becomes important. In addition, in the case of using mimosine as a bioherbicide, knowledge of its biosynthesis is necessary to increase its content in *L. leucocephala* or in other plants.

### 4.3. Terpenoid Biosynthesis

The main components of the *Eucalyptus* essential oils are monoterpenes and sesquiterpenes [222]. The biosynthesis of plants is formed in the cytosol of the mevalonic acid pathway, while monoterpenes are formed in the plastids of the methylethritol phosphate pathway [275]. However, there may be competition for the phenyl pyrophosphate substrate between mono and sesquiterpenes [276]. The involved terpene synthases have been divided into seven sub-families [277]. The genes related to the terpenoid biosynthesis began to be extensively studied only in recent years. Among sequenced angiosperm plant genomes, *Eucalyptus* has the highest number of terpenoid biosynthetic genes [278]. In total, 106 and 113 putative terpene synthase genes were identified in *E. globulus* and *E. grandis*, respectively—approximately four times as many as in *Arabidopsis thaliana* and twice as many as in *Vitis vinifera* [279]. Most of these genes were found in large (up to 20 genes) genomic clusters. This interesting pattern of clustering of biosynthetic genes for some allelochemicals (e.g., benzoxazinoids, cyanogenic glucosides, terpenoids, and alkaloids) in chromosomes has been found recently [280]. Supposedly, it promotes stable inheritance of functional chemical defense pathways in populations [281]. About 30 examples of clusters of genes encoding products important for secondary metabolic pathways were reviewed recently [282], but only for grass species.

In other woody plants rich in terpenoids, the terpene biosynthesis genes were found to be less frequent. In the *Cinnamomum camphora* transcriptome 67 unigenes likely involved in terpenoid biosynthesis were identified [283]. Data on the biosynthesis of terpenes in conifers is much limited. Mao et al. [284] identified 372 unigenes involved in the oleoresin (viscous mixture of terpenoids) biosynthesis in loblolly pine (*Pinus taeda)*, but only 74 of them are involved in the terpenoid backbone biosynthesis.

The terpene synthases control final products of the terpene cyclisation, and differential expression of its genes can reflect the differences between chemotypes (variants in chemical composition of essential oils) [276]. Three distinct chemotypes were identified in *Eucalyptus tricarpa*: with dominance of monoterpenes (>80% of all terpenes) or sesquiterpenes (>55%), and low-to-medium proportions of sesquiterpenes (10–55%) [276]. An even narrower classification of terpene chemotypes (for monoterpene compounds) may exist in *E. grandis*. Either α-pinene or 1,8-cineole dominate among monoterpenes. They originate from different carbocations and are products of different terpene synthase genes [279]. At least five different chemotypes, including linalool-, borneol-, camphor-, cineole-, and nerolidol-types have been identified in *Cinnamomum camphora* L., which has allelopathic activity, and all parts of which are rich in essential oil [285]. A metabolic analysis and transcriptome sequencing of *C. camphora* were done relatively recently, and it was shown that terpene synthase and oxidoreductase activities could explain the differential accumulation of terpenoids between the two chemotypes [283].

The significant dependence of eucalypts on both amount and composition of the essential oils implies a great importance of regulatory genes in biosynthesis of terpenes. This is in agreement with the fact that many transcription factors are also located close to clusters of terpene synthase genes [279]. The terpene emission in *Eucalyptus* is known to come mainly from leaves, which have numerous the sub-dermal secretory cavities (glands), but also from other terrestrial parts, for example, from flowers to attract pollinators. This was confirmed by Kulheim et al. [279], which showed that the largest proportion of the *E. grandis* terpene synthase genes was highly expressed in “green tissues” (mature and young leaves, floral buds, and shoot tips). The authors also discovered root-specific cluster of the terpene syntase genes, but the role of terpenes in these tissues remains unknown. He et al. [250] demonstrated that there were no volatiles in soil water from root exudates produced by the laboratory plants and in the field samples.

A large number of different terpenes and erpene synthase genes open a broad way for metabolic engineering of the composition of the essential oil, but at the same time make it difficult. To understand the reasons for the differences between the chemotypes of eucalyptus and other species with a complex composition of allelochemicals, it is necessary to study the expression of their biosynthetic and regulatory genes. For example, root-specific terpene syntase genes, recently discovered in *E. grandis* [279], can also be found in other trees.

## 5. Natural Biopesticides Originated from Trees

### 5.1. Allelochemicals as Herbicides

Among all crop losses caused by abiotic and biotic environmental factors, weeds are inflicting the most damage (34% on average), actually the same amount as pests and pathogens combined [286]. The world economic losses due to weeds are estimated to be more than US $ 100 billion dollars [287]. Until about the middle of the 20th century, physical (mechanical or hand weeding) action was the main method of fighting weeds, but then it was changed to chemical action, which significantly reduced labor.

In the 2010s, around four million tons of pesticides were used annually in the world [288], and herbicides accounted for up to half of this volume. Many modern herbicides are selective towards particular crops and are used in small doses. However, the problem lies in the fast evolution of herbicide resistance in weeds and threats to environment, human, and animal health. In addition, organic agriculture being a prominent direction and becoming increasingly popular lately does not generally imply the use of synthetic pesticides, including herbicides; and that is why weed control still remains rather problematic. Thus, development of new and safer herbicides for both traditional and organic agriculture is still remaining an important task nowadays. However, commercial herbicides have only approximately 20 modes of action, and no new modes of action were introduced in over 25 years [289].

However, Nature is a rich source of diverse bioactive compounds that could be used as an active ingredient for new herbicides. Unfortunately, this source is not used sufficiently, only 8% of conventional herbicides are derived from natural compounds [290]. For example, non-selective phytotoxins phosphinotricin and bialaphos produced by the soil bacteria *Streptomyces hygroscopicus* and *S. viridochromogenes* are widely used as active ingredients of herbicides [289]. This substance is largely employed in plant genetic engineering as a selective agent.

Allelochemicals that possess phytotoxicity in nature could act as potential natural bioherbicides. Compared to synthetic herbicides, they have several advantages: (1) they exhibit structural diversity and possess complex structures, (2) they are safe, as they quickly decompose, and (3) they have a different mode of action [291]. In addition, they might be used together with synthetic herbicides that could reduce probability of the herbicide resistance development in weeds and decrease dose of synthetic herbicides while maintaining the same efficiency [192]. From an ecological perspective, secondary metabolic compounds can be considered as chemical weapons to ward off predators and competitors for limited resources and contribute to explaining the ecological impact of their hosts (for instance, *A. altissima*) on ecosystems [292].

Allelopathy could be employed in several ways for weed control [293,294]: (1) via crop rotation, (2) as ground cover species, (3) application of plant residue on surface (mulching) or deep in the soil (green manure), (4) application of allelopathic water extracts, and (5) as new natural bioherbicides formulated from allelochemicals. Due to the long duration of the woody plant cultivation, direct use of the first two methods for weed control is not feasable, and only the last three of them are applicable. The first step in search for potential bioherbicides candidates is to evaluate the phytotoxic activity of vegetable tissue extracts in a laboratory.

**Figure 16:**
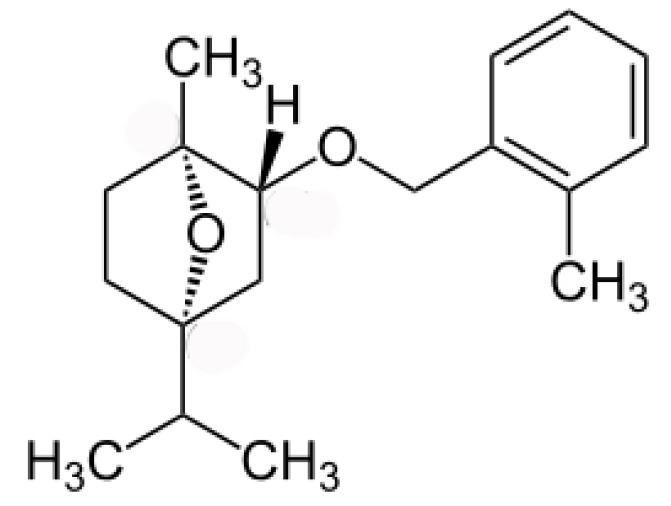
Cinmethylin.

Essential oils of plants, including eucalypts, appear to be good candidates as bioherbicides because of their availability and low cost [295]. The herbicide cinmethylin (Figure 16) was developed by Shell Chemical Comp. based on the monoterpene 1,4-cineole (1-methyl-4-(1-methylethyl)-7-oxabicycloheptane) [296]. 1,4-Cineole is a structural isomer of 1,8-cineole, but it is less abundant component of plant essential oil [297]. Cinmethylin is a 2-benzyl ether substituted analog of 1,4-cineole, where a benzyl ether moiety is added to decrease the volatility of the cineole ring by several orders of magnitude, thereby rendering it more suitable for herbicide use [298]. Cinmethylin was commercialized in 1982 under the trade names of Argold and Cinch and is used in the transplanted rice against grass weeds at low application rates of 25 to 100 g a.i./ha [299].

**Figure 17:**
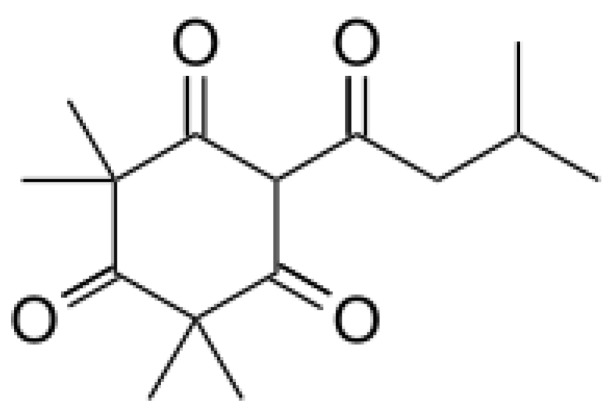
Leptospermone.

**Figure 18:**
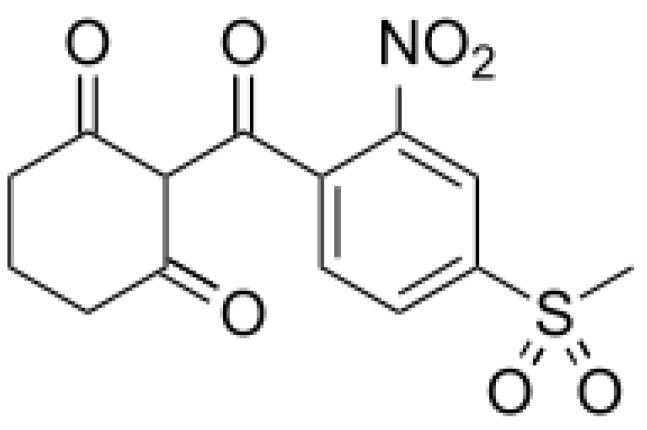
Mesotrione.

**Figure 19:**
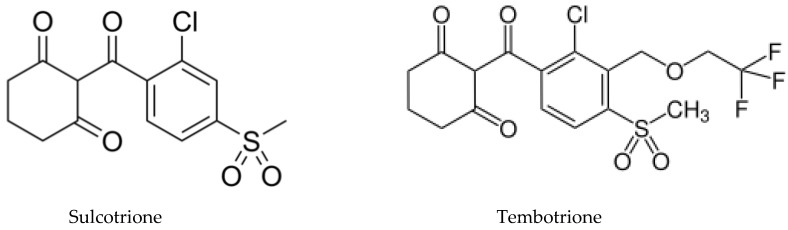
Leptospermone derivatives.

Leptospermone (1-hydroxy-2-isovaloryl-4,4,6,6-tetramethyl cyclohexen-3,5-dione, Figure 17) isolated from one of the plants of the *Myrtaceae* family, i.e., *Callistemon citrinus* Curtis, is another allelochemical used as a herbicide. It is a natural triketone inhibiting *p*-hydroxyphenylpyruvate dioxygenase (HPPD) enzyme that leads to disruption in carotenoid biosynthesis and loss of chlorophyll [300]. This was the most recently discovered and introduced new herbicidal mode of action for commercial herbicides [301]. Since pure leptospermone was used at very high concentrations (9000 g/ha), it was used as the basis for synthetizing an analogue, mesotrione (Figure 18), which was becoming efficient at 75–225 g/ha concentrations [8], as well as sulcotrione and tembotrione (Figure 19). Mesotrione sold by Syngenta AG under the Callisto brand is used as a pre- and post-emergence herbicide in corn cultivation [170]. No weeds have evolved resistance to HPPD inhibitors yet, but transgenic plants with resistance to this herbicide were generated [302]. Later, it was also discovered that large quantities of leptospermone are contained in the essential oils of *Leptospermum scoparium* J.R., G. Forst, allelopathic and invasive shrub originating from Australia and New Zealand [303]. McCoy et al. [267] suggested that juglone is a prime candidate for developing a novel bioherbicide, because its mode of action appeared to be distinct from that of any existing synthetic commercial herbicides.

Natural compounds, as a rule, cannot be used in a pure form as pesticides due to their insufficient stability, activity, or selectivity. For example, monoterpenes are generally too volatile to be used directly as herbicides [233]. Therefore, the most promising way is to modify their chemical structure to enhance pesticidal properties [304].

Both of the above-described herbicides are based on allelochemicals from woody plants and were obtained by modifying them: cinmethylin—by reducing volatility of the 1,4-cineole and mesotrione—by increasing the leptospermone activity. In order to reduce volatility, but preserving or increasing phytotoxicity of 1,8-cineole, the most common component of the *Eucalyptus* essential oil, its hydroxy and ester derivatives were obtained [305]. Laboratory pre-emergence bioassays demonstrated that these substances inhibited germination and growth of *Lolium rigidum* and *Raphanus sativus*. The increased lipophilicity of the carboxylic acid portion of cineole ester derivatives did not affect the herbicidal activity, and they could be considered as environmentally acceptable herbicides [305]. Additional experiments showed that reduced root and shoot growth effect of cineole derivatives were due to post-emergence activity rather than delayed germination [306]. Phytotoxicity of ester derivatives may be due to metabolic cleavage of esters to hydroxy cineole and carboxylic acid within the plant [306]. There is also another study that demonstrated herbicidal activity of propionate derivatives of mimosine against growth of *Brassica rapa*, but they were less efficient than original mimosine [204]. Recently, Duran et al. [307] evaluated *O*-acyl and *O*-alkyl derivatives of juglone with different linear chain lengths on wheat, four target and four weed species. It was shownd that lipophilicity and functional group were responsible for their activity change. *O*-acyl derivatives were more active when changes were introduced at positions 5 and 2 in the naphthoquinone scaffold. As a result, three prospective 5-*O*-acyl juglones were selected for the further development of bioherbicides [307]. The ailanthone derivatives have long been studied in medicine [308], but their herbicidal activity has not been reported. An important factor in enhancing the herbicidal activity of allelochemicals can be various additives. For instance, the addition of a wetting agent to the *Wollemia nobilis* extract increased the suppression of *Lolium rigidum* as much as a 4-fold increase in the extract concentration [160].

Selectivity of action is an important property of herbicides, but it is rarely observed with substances of natural origin. It is known that citronellal is more effective against broad-leaved weeds [233], while cineole is more effective against grassy weeds [235]. Selective effects of cineole derivatives on dicotyledonous and monocotyledonous species were demonstrated for 2-*endo*-hydroxy-1,8-cineole that showed the highest activity against *Raphanus sativus*, and for 3-*exo*-hexoxy- 1,8-cineole—against *Lolium rigidum* [306].

The cost of raw materials for the production of active ingredients of herbicides should be relatively cheap, and this requirement is well met by the components of the eucalyptus essential oils, which in addition to isolation from natural raw materials can be obtained by chemical synthesis. This synthesis is difficult for mimosine and ailantone, but they are contained in relatively large quantities in the biomass of fast-growing trees, from where they can be easily extracted. Since juglone is synthesized in the phylloquinone pathway, green algae, cyanobacteria, or certain menaquinone-synthesizing bacteria can be used for its large-scale production [267]. Recent studies have shown activity of allelocochemicals of *Ailanthus altissima* [38], *Cinnamomum camphora* [309], *Eucalyptus* [50], and *Juglans* [195] against cyanobacteria that causes algae blooms, and, therefore, they can be potentially used as algaecides.

Use of the plant residues of allelopathic plant species is especially prospective in organic farming. Mulch could limit the growth of weeds both due to physical characteristics (preventing exposure to light, reducing available moisture, etc.) and its allelopathic properties. Several invasive trees, e.g., *A. altissima*, are especially appropriate plant species for using as potential source of mulch materials due to both their allelopathic properties and frequency in which they are removed following management activities [291]. Incorporation of plant residues into the soil as a bioherbicide green manure allows not only to fight weeds, but also ensures reduction of soil erosion and amelioration of soil physical properties, increases of soil organic matter and nutrient retention, thus reducing dependence on mineral fertilizers [310]. At the same time, studies have shown that the litter of such allelopathic species as *Acacia holosericea* [311], Chinese fir ([140], and *Eucalyptus saligna* [62] prevented the growth of the understorey species physically, and not chemically. It is possible that the litter, accumulated and slowly decomposed (especially in conifer species), is responsible for reduced natural forest regeneration.

### 5.2. Allelochemicals as Insecticides

**Figure 20:**
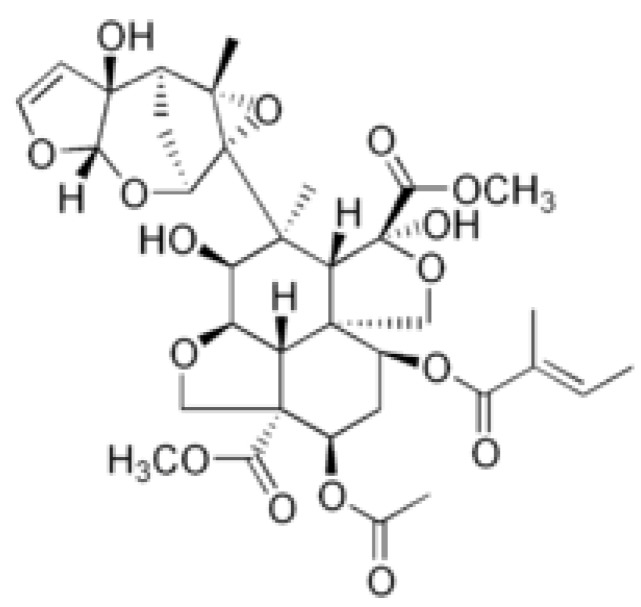
Azadirachtin.

Allelochemicals from tree species also could be used as insecticides. Among four main types of botanical products used for insect control—pyrethrum, rotenone, neem, and essential oils [312], the latter two are obtained from woody plants (fully and partially, respectively). Among them, the most famous is limonoid triterpene azadirachtin discovered in the 1960s (Figure 20) and extracted from the seeds of the Indian neem tree [*Azadirachta indica* A. Juss (Meliaceae)] [313]. In 1994, the EPA registered a botanical insecticide containing azadirachtin, and a number of commercial preparations are currently produced where it is an active ingredient. Azadirachtin is a system pesticide and has two effects on insects: it blocks the synthesis and releases molting hormones (ecdysteroids) leading to incomplete ecdysis in immature insects or to sterility in adult female insects, and it is a potent antifeedant [312]. Azadirachtin is nontoxic to mammals, birds, fish, and pollinators, as well it has a short half-life and popular in the organic agriculture. In forestry azadirachtin demonstrated its efficiency in protecting ash trees from emerald ash borer (*Agrilus planipennis* Fairmaire)—an exotic invasive pest that threatens *Fraxinus* spp. trees throughout North America [314]. Results support the use of azadirachtin as an environmentally acceptable systemic insecticide in urban [315] and in ecologically sensitive [316] environments, such as riparian and source-water forests, wooded wetlands, or conservation areas.

*Azadirachta indica* contains about 200 allelochemicals in its different parts [317]. It was shown that bark and leaf extracts inhibit germination and growth of crops and weeds in bioassays [99,318], and the effect was associated with phenolic compounds [99]. Later, two potent growth inhibitory substances, nimbolide B and nimbic acid B, were isolated from leaf extracts, and the effectiveness of these compounds on the dicot plant *Lepidium sativum* L. was several times greater than that on the monocot plant *Echinochloa crus-galli* [98]. Azadirachtin were not phytotoxic to the herb species in the greenhouse [319] or to grape in the field [320].

**Figure 21:**
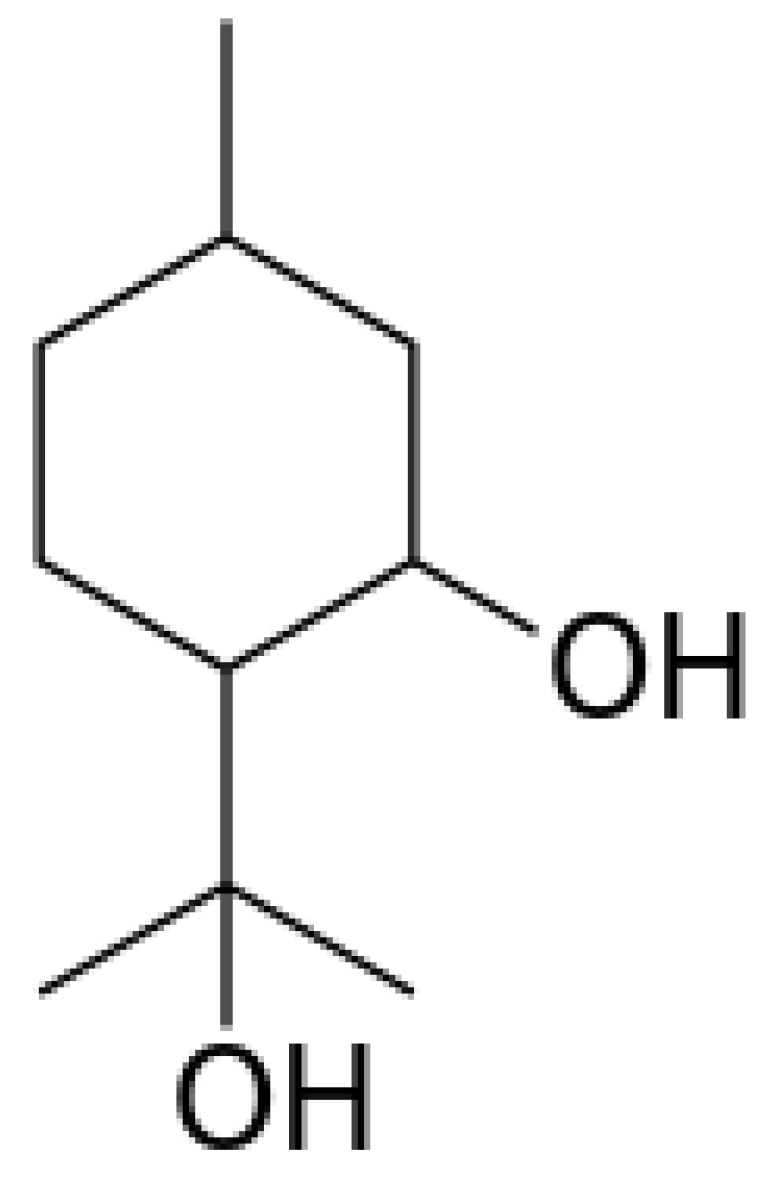
*p*-Menthane-3,8-diol.

An important group of natural insecticides are essential oils. Numerous studies have shown insecticidal activity of essential oil from various eucalypt species [223,321]. In 1948, the eucalypt oil has been registered as an insect repellent in the USA, and now some commercial repellents are available [222]. The *p*-menthane-3,8-diol (Figure 21), similar to DEET, extracted from the leaves of lemon eucalypt, *Eucalyptus citriodora*, has stood out among them for its high efficiency and was registered as a biopesticide repellent by EPA in 2000 [322]. *p*-Menthane-3,8-diol has a lower vapour pressure than other highly volatile active ingredients of ssential oils and provides very high protection from insects over several hours, whereas the essential oil is effective only for around one hour [323]. Numerous studies have indicated that essential oils of *Cinnamomum camphora* have activity against stored product [324] and agricultural [325,326] pests, and other insects [107].

*A. altissima* is resistant to insect pests due to the high content of various secondary metabolites in its tissues. This was noticed a long time ago: for example, this plant has been used to control agricultural pest in China [165]. The leaf extract of *A. altissima* demonstrated low insecticidal activity against yellow fever mosquito larvae (*Aedes aegypti*) [327]. De Feo et al. [292] have shown that aqueous leaf extract demonstrated higher mortality compared aqueous root extracts, but the ailantone demonstrated highest aphid mortality. Thus, the insecticidal activity of ailanton is of interest for development of biopesticides.

Ishaaya et al. [328] demonstrated that mimosine inhibits growth and development of *Tribolium castaneum* decreasing the activity of trehalase, invertase, and amylase. Nquyen et al. [329] has first synthesized novel amino alcohols and phosphoramidothionate derivatives from mimosine and evaluated their activity against termites. Mimosinol and deuterated mimosinol (D-mimosinol) had much higher (3040– times) insecticidal activity than mimosine, which could be a result of tyrosinase inhibition, where as two phosphoramidothionate derivatives of mimosinol were up to 100 time more active than mimosine (comparable to the commercial insecticide rotenone), which may be attributed to the acetylcholinesterase inhibition. Such high activity makes mimosine derivatives potential candidate for novel bioinsecticides.

Juglone toxicity for various insects has been shown in various studies [330,331], although some lepidopteran species were resistant, such as *Actias luna* that possesses high activity of quinone reductase and was able to detoxify juglone [332]. The mechanism of insecticidal activity of juglone has not fully understood. Mitchell and Smith [333] assumed that juglone may inhibit activity of ecdysone 20-monooxygenase, an enzyme responsible for converting the molting hormone ecdysone. Magiri et al. [334] has showed that juglone inhibited respiration in the *Glossina morsitans* mitochondria. Using metabolomics analysis, it was demonstrated that the juglone caused a disturbance of the *Apis gossypii* physiology by affecting its hemolymph metabolomic profile [335]. Finally, Hu et al. [336] found that juglone inhibits phenoloxidase activity in haemolymph of larvae of *Pieris rapae* Linne and *Helicoverpa armigera* Hübner. In insects, phenoloxidase plays an important role in the developmental processes of immunity defence, and its inhibitor has a perspective as a bioinsecticide. In addition, studies have been also conducted to evaluate the toxic activity of juglone derivatives. Evaluation of substituted hydroxyquinones, their salts and halogenated quinines on *Aedes aegypti* L. has showed that three substances are several times more effective than juglone, and activity of 3-bromojuglone is similar to the temephos pesticide [337]. These results indicated that the bromo substituent, in bromoquinones, is highly reactive and may be used as efficient biocontrol agent against the mosquito larvae.

Another substance of quinone nature, tectoquinone (β-methyl anthraquinone), isolated from *Cryptomeria japonica* D. Don, showed a mosquito larvicidal activity against *Aedes aegypti* and *A. albopictus* [338]. It is believed that tectoquinone is responsible for the resistance of teak wood (*Tectona grandis* L.f.) to insect attacks [339].

### 5.3. Development of New Biopesticides

Identification of allelochemicals is of utmost importance for the development of new pesticides, and by now quite a lot of phytotoxic compounds are known. However, despite of all advantages of using natural substances as bioherbicides, there is a number of problems that prevent commercial use thereof. First, a relatively low activity associated with a low specificity should be addressed. In contrast to synthetic herbicides, which are usually targeted at a specific stage of metabolism, allelochemicals tend to affect simultaneously many physiological processes, but none of them is extremely affected [10]. Juglone, which main mode of action is associated with oxidative stress could serve as an exception. Second, allelochemicals are usually present in plants at low concentration and as a rule act synergistically, and a purified allelopathic compound may act on target plants with much higher or much lower strength [8]. Third, such allelochemicals as essential oils contain dozens of components, which composition and concentrations depend on many factors including season, climate, age, geographic region, plant genetics, etc. [340]. Thus, the drawback of preparations based on natural substances could be variability of their composition and accordingly efficiency depending on the state of raw materials. Fourth, the reverse side of bioherbicides environmental friendliness (rapid decomposition) is their relative instability in the environment. Thus, it is most appropriate to use natural allelochemicals as the basis for developing new synthetic herbicides, but their chemical structures are often too complicated, and its synthesis at the industrial scale turns out to be extremely expensive [341]. Finally, registration of new products includes very expensive regulatory approval procedures that would not be compensated for by the small profits from the limited use of these pesticides under specific environmental conditions and requirements (for example, in greenhouses or in organic farming) [342]. This is very similar to a situation with transgenic plants. The main market share is occupied by crops grown on tens of millions of hectares (soybean, corn, cotton, rapeseed, etc.), while less common plants, such as horticultural plants, are not being commercialized since the cost of their registration would not be compensated for by profit obtained from growthing them.

However, many significant studies demonstrated that allelopathy possesses promising potential for use in agricultural production for economical and environmentally friendly weed management in agricultural systems. When developing new herbicides based on allelochemicals, extensive field trials are required to study various aspects of interaction thereof both with physicochemical properties of soil and soil microorganisms. Studies on the allelochemicals biosynthesis were started only recently, and it is necessary to expand work in this direction, as well as on transport and mode of action that could be used in future for improving allelopathic potential of trees using modern plant breeding techniques. For instance, little is known about production, storage, and movement of the ailanthone in the *A. altissima* tree [13], and we are unaware of any publications on the ailanthone biosynthesis genes. In addition, there are prospects in using plant residues of allelopathic trees as a mulch, as well as combined herbicides (at reduced rates), and allelopathic extracts. Although many allelochemicals were identified, only a few bioherbicides based on them were commercialized, which requires intensifying research on their chemical modification in order to improve their properties. The growing interest to environmental protection and organic agriculture in the world will contribute to the development of new pesticides based on natural allelochemicals.

## 6. Transgenic Trees and Allelopathy

Due to the great economic importance of eucalypt - this is the main plantation tree - intensive research is being carried out on production of transgenic eucalypts. According to the Cartagena Protocol on Biosafety, GMOs planted or released into the environment should not have any potential adverse effects on biodiversity [343]. Thus, transgenic plants should also be evaluated for their allelopathic activity including transgenic eucalypts containing the *codA* gene that increases salt tolerance. The *codA* gene was isolated from the soil bacterium *Arthrobacter globiformis* and encodes choline oxidase enzyme that has no known direct allelopathic effects [344]. However, transgenic plants are more tolerant to salt stress, and it is known that stressful conditions can change both the sensitivity to allelochemicals and their production.

Initially, the effects of *codA* on allelopathic activity of transgenic *Eucalyptus camaldulensis* [345] or *E. globulus* [346] were tested under net-house conditions. In addition, long term trials were carried out for transgenic *E. camaldulensis* [347] and *E. globulus* [346] in Japan. Authors had evaluated the allelopathic effects of eucalypts on test species *Lactuca sativa* using sandwich and soil mix methods, and no significant differences were found between the transgenic and non-transgenic plants. Kikuchi et al. [345] also demonstrated no change in the qualitative composition of the volatile substances and the phenolic compounds in transgenic eucalypts compared to control plants. Later Gilani et al. [348] studied allelopathic effects of essential oils from transgenic *E. camaldulensis* with another salt resistance gene, mangrin, on seed germination and early growth of *L. sativa* L. The results showed no significant inhibitory effects of transgenic plants. 1,8-Cineole and α-pinene were tested as major oil constituents of *E. camaldulensis*, and no variation was found between transgenic and non-transgenic lines. These results revealed that eucalypts that contained salt tolerance genes did not change their allelopathic activity.

The eucalypt was the first example of metabolic engineering of a woody essential oil plant. In 2010 Ohara et al. [349] transformed the *Eucalyptus camaldulensis* Dehnh. with constructs containing limonene synthase gene for localization of the enzyme either in the cytosol or in plastids. The plastidic and cytosolic expression of transgene yielded 2.6- and 4.5-times more limonene in leaf extracts than that accumulated in non-transgenic plants, respectively, but had only a small effect on the limonene emission from the leaves. Surprisingly, the limonene synthase expression induced the accumulation of 1,8-cineole and α-pinene, although formation of these two majour monoterpenes of *Eucalyptus* is catalysed by independent monoterpene synthases [349]. However, an assessment of the allelopathic activity of these plants has not been reported.

Evaluation of transgenic eucalypts generated for commercial use also showed no changes in their allelopathic activity. The *E. grandis × E. urophylla* hybrid was transformed with either the *endo*-1,4-β-glucanase (*cel1*) or the cellulose-binding protein A genes to improve growth rate and wood quality [350]. The analysis of yield or composition of essential oil from leaves harvested in 2009–2010 demonstrated that there were no significant differences between transgenic and non-transgenic trees in the field trials. In 2015, Brazil approved commercial use of the transgenic eucalypts with the *cel1* gene developed by FuturaGene. Another *E. grandis × E. urophylla* hybrid line has been modified for freeze tolerance and sterility, which should not increase chemical exudates and leaching, by ArborGen (USA). Field trials confirmed the lack of evidence for allelopathic effects of transgenic plants on a variety of grasses and broad leaf weeds in test plots [351].

Significantly fewer studies have been conducted on other transgenic trees with high allelopathic activity, in particular *Leucaena leucocephala*. Genetic transformation of *L. leucocephala* with o-methyltransferase gene in antisense orientation resulted in reducing lignin content, increasing cellulose content, and was accompanied by an increase in methanol soluble phenolics [352]. Jube and Borthakur [353] first reports about reducing the toxicity of a tree-legume using a bacterial gene for degradation. Especially with the aim of reducing the content of mimosine allelochemical *L. leucocephala* was transformed with two genes, *pydA* and *pydB*, encoding a meta-cleavage dioxygenase (EC 1.13.11.2) and a pyruvate hydrolase (EC 3.7.1.6), respectively, from the mimosine-degrading *Leucaena* symbiont *Rhizobium* sp. strain TAL1145. The mimosine contents of the *pydA*-expressing lines were reduced up to 22.5% in comparison to the wild-type, but no changes were observed in the *pydB*-expressing lines.

In addition to *Eucalyptus* and *Leucaena*, there are also isolated reports on the assessment of allelopathy in transgenic plants of other tree species that do not have significant allelopathic activity. This is due to the fact that genetic transformation can cause a so-called unforeseen changes unrelated to the nature of the gene transferred. Allelopathic tests on *Lactuca sativa* showed that the root exudates and leaf litter of field-grown transgenic *Populus alba* with xyloglucanase gene for increasing the cellulose content did not produce harmful substances [354]. Guo et al. [282] evaluated the allelopathic activity of leaves from *Populus tomentosa* with the *DREB* transcription factor under field conditions. Sandwich and the soil-mix methods with *Lactuca sativa* seeds did not show significant differences between the transgenic and non-transgenic lines. At last, transgenic American chestnut (*Castanea dentata*) expressing an oxalate oxidase gene for fungal disease resistance was evaluated for potential allelopathy using five native species in its traditional habitat (grass, forb, shrub, coniferous tree, and deciduous tree) [355]. Seed germination or total biomass of seedlings in the greenhouse were not significantly different in transgenic and non-transgenic leaf litter. From the data available today, it can be concluded that neither the targeted alteration of genes associated with allelochemical activity nor unforeseen changes in transgenic woody plants led to a change in their allelochemical activity.

RNA interference-mediated down-regulation of the 4-coumarate: coenzyme A ligase in *Populus tremula* alters lignification and plant growth [356] and rhizogenesis [357]. Metabolomic studies of these plants revealed strong changes in the biosynthesis of phenolic compounds, in particular the accumulation of glycosylated forms, which may indicate their cytotoxicity. It was also noted that lines with a high accumulation of glycosylated forms of phenolic compounds showed reduced growth (our unpublished results). Thus, suppression of lignin biosynthesis genes has affected the phenolic metabolism of transgenic aspen plants, which could change their allelopathic activity.

Although Coder and Warnell [12] have placed birch in a group of trees with slight allelopathic effects, a significant impact of genotype and ontogeny on birch shoot secondary chemistry was shown [358]. It was shown that elevated CO_2_ and O_3_, characteristic of global climate change, increased phenolic compound contents in leaves of *Betula pendula* Roth [359]. In addition, birch is one of the main hardwood species in boreal forests and promising for establishment of forest plantations including transgenic genotypes. It is not excluded that genetic transformation, especially affecting the primary metabolism pathway, can lead to an increased level of allelochemicals. In order to increase productivity, we transferred the *GS1* gene from *Pinus sylvestris*, encoding cytosol form of glutamine synthetase, main enzyme of nitrogen metabolism in plants, in *Betula pubescens* and *B. pendula* [360]. The transgenic birch plants had an elevated content of glutamine, as well as glutamic and aspartic acids, and rooted more rapidly than the control plants due to increased auxin levels [361]. In addition, open-air tests demonstrated that birch plants expressing the *GS* gene use nitrogen more efficiently under nitrogen deficiency conditions [362]. It can be assumed that altered levels of amino acids and auxins will lead to a change in the level of allelochemicals. Moreover, forest plantations are usually establsihed on poor soils, and the cultivation of plants in conditions of lack of nutrients is stressful. It is possible that such stress will increase both the release of allelochemicals by transgenic birch trees, and sensitivity to them in the surrounding vegetation.

The transgenic approach can also be used to study the allelopathic activity of plants. So far, it has been applied only on grassy plants. For example, using knock-outs of the relevant diterpene synthases, Xu et al. [363] demonstrated that rice momilactones are involved in allelopathy including suppressing growth of the widespread rice paddy weed, barnyard grass (*Echinochloa crus-galli*). Plants with this alteration were not allelopathic. Thus, these results not only provide novel genetic evidence for natural product-mediated allelopathy, but also furnish a molecular target for breeding and metabolic engineering of this important crop plant. Later, RNA interference approach was used for suppression of phenylalanine ammonia-lyase gene in rice genotype with high allelopathic potential [364]. Transgenic plants demonstrated the lower concentrations of phenolics in the root tissues and root exudates and reduced phytotoxicity against *Echinochloa crus-galli*.

The use of woody plants as models for the study of allelopathy has not been reported until recently. To facilitate allelopathic research, Stanisic et al. [365] has first established apple transgenic hairy root cultures as a new tool for allelopathic assays in 2019. Authors transformed four apple cultivars by *Agrobacteium rhizogenes* and demonstrated the phytotoxic effects of hairy root exudates on shoot and root development and growth of *Arabidopsis thaliana* seedlings. Genetic transformation did not disturb secondary metabolite production in apple, and untransformed and transgenic root tissues had similar content of phenolic acids and flavonoids. As putative allelochemicals chlorogenic and caffeic acids and dihydrochalcones phloridzin and phloretin were identified [365]. Obtaining a hairy root culture for plants with high allelopitic activity will allow a better study of the biosynthesis of these compounds and explore the possibilities of genetic manipulation of these processes.

One of the directions is production of allelochemicals in bioreactors using plant cell cultures, but this technology is now economically justified almost exclusively only for the production of valuable pharmaceutical compounds. One of the most effective strategies for enhancing the biotechnological production of secondary metabolits is elicitation [366]. Biotic and abiotic elicitors stimulate plant defense, including allelopathic, but for the application of this technology it is necessary to know in response to which factors plants stimulate production of allelochemicals. However, information on this topic in woody plants is rather limited.

Genetic engineering techniques can be used to enhance allelopathy of agricultural plants as a means of controlling weeds. This can be achieved by increasing their production in plants or changing the localization of their expression (for example, increasing the content in the roots) or seasonality. Their synthesis in plants where they were absent earlier is less likely. This direction requires the identification of genes that encode enzymes involved in synthesis of powerful allelochemicals and find out how expression of these genes is regulated.

The use of classical traditional breeding methods to increase allelopathy is still acceptable for herbaceous plants, but less efficient for trees. Modern biotechnology methods such as gene transfer or genome editing can be used. However, it is unlikely that trees with a complex composition of allelochemicals, such as eucalyptus, will be purposefully modified to change their allelopathic potential. As already shown, dozens of genes are involved in the synthesis of terpenes, and this process is still dependent on various external and internal factors. It is more likely that this will occur as a side effect of solving other, more economically feasible tasks, such as a change in the composition or yield of the essential oil. Purposeful change in allelochemical potential has a higher probability of use on species containing one major alleochemical, such as mimosine or juglone. To perform these tasks, it is necessary to understand genes involved in regulation and biosynthesis of allelochemicals. It may be necessary to obtain transgenic model plants to study these processes.

## 7. Conclusions

In recent years, great progress has been achieved in study of allelopathy of woody plants. In addition, a number of new allelochemicals has been identified. Recent advances in genomics, transcriptomics, proteomics and metabolomics have played an important role in deciphering the metabolic pathways for a number of phytotoxins. However, in general, there are still not enough data on their biosynthesis, transport, excretion systems, and changes in the soil. The genetics of allelopathic processes in woody plants has been poorly researched. Studies conducted on biotechnological areas, obviously, are not enough. The wide distribution of the forest plantations, especially for species with pronounced allelopathic potential, such as eucalypt and acacia, and in new areas requires a comprehensive study. It is necessary to take into account the influence of various factors on the qualitative and quantitative allelochemical compositions. Such interactions as synergism and antagonism between different allelochemical substances should be evaluated. It is also necessary to use field tests and modern molecular research methods more widely. As for the forest ecosystems, the previously defined concept of allelopathy as a localized interaction of plants and plants through the release of allelochemicals has been expanded in recent years to a phenomenon at the ecosystem level. Therefore, it is necessary to apply modeling techniques taking climate change into account. These areas should be at the center of future research.

## Figures and Tables

**Table 1:** Allelopathy of angiosperm tree species.

Allelopathic Species	Test Species ^a^	Analysis Type	Compounds	Reference
*Albizia adianthifolia, Buddleja saligna, Combretum kraussii, Halleria lucida, Rapanea melanophloeos, Vachellia sieberiana*,	*Lactuca sativa*	laboratory bioassay	leaf extracts	[91]
*Albizia lebbeck*	crops (5)	laboratory bioassay	leaf extracts	[92]
*Alstonia scholaris*	*Lactuca sativa*, crop (1), weed (1)	laboratory bioassays, pot culture	leaf and litter extracts, leaf and litter powder, allelochemicals	[93]
*A. scholaris*	weed (1)	laboratory bioassays	leaf and bark extracts	[94]
*Ailanthus altissima, Ulmus pumilla, Robinia pseudoacacia, Populus alba*	grasses (6)	laboratory bioassays	leaf litter extracts	[95]
*A. altissima, Robinia pseudoacacia, Fraxinus angustifolia, Populus alba*	*R. pseudoacacia*, *P. alba*, grasses (11)	laboratory bioassays	leaf litter extracts	[96]
*Amorpha fruticosa, Hedysarum mongolicum, Sabina vulgaris, Hippophae rhamnoides*	shrub (1)	laboratory bioassays	leaf extracts	[97]
*Azadirachta indica*	weeds (4), crops (3), grass (1)	laboratory bioassay	leaf extracts, allelochemicals	[98]
*A. indica*	crops (6), weeds (3)	laboratory bioassay	bark and leaf extracts	[99]
*Cabralea canjerana*, *Carapa guianensis, Cedrela odorata, Hortia oreadica, Spiranthera odoratissima, Toona ciliate, Zanthoxylum petiolare*	*Lactuca sativa*, crops (3)	laboratory bioassays	allelochemicals	[100]
*Castanea dentata*	*Lactuca saliva*, trees (6)	laboratory bioassay	leaf extracts	[101]
*Castanea dentata*, *C. mollissima*	*Lactuca sativa*, crops (5)	laboratory bioassays	leaf extracts	[102]
*Casuarina equisetifolia*	crops (1), weed (1)	laboratory bioassay	fog-drip leachates; needle, litter, cones and soil extracts	[103]
*C. equisetifolia*	trees (3)	laboratory bioassays	root, litter and soil extracts	[104]
*C. equisetifolia*	*C. equisetifolia*	laboratory bioassays	allelochemicals	[105]
*Cinnamomum camphora*	crop (1)	pot culture	leaf powder, allelochemical	[106]
*C. camphora, C. glaucescens, C. tamala*	*Lactuca sativa*, grass (1)	laboratory bioassays	leaf, root and fruit essential oils	[107]
*C. septentrionale*	tree (1)	pot culture	leaf litter	[108]
*Cinnamomum septentrionale*	crop (1)	pot culture	leaf litter	[109]
*Diospyros kaki*	*Lactuca sativa*, crops (5)	laboratory bioassays	leaf extracts	[110]
*Lonicera maackii*	*Lonicera maackii*, grasses (5)	laboratory bioassay	leaf extract	[111]
*L. maackii*	*Arabidopsis thaliana*	laboratory bioassay	leaf extract	[112]
*Lupinus jaimehintoniana*	*Lactuca sativa*	laboratory bioassays	leaf, seed, shoot and phloem extracts	[113]
*Morus alba*	crops (1), weeds (1)	laboratory bioassay, pot culture	leaf extract	[114]
*Nerium oleander*	weeds (1)	laboratory bioassay, pot culture	leaf extract	[115]
*Nyssa yunnanensis*	*N. yunnanensis*	laboratory bioassays, greenhouse, field	extracts, soil, litter	[116]
*Paulownia tomentosa, P. elongata × P. fortunei*	crop (1), grasses (2)	laboratory bioassays	leaf extracts	[117]
*Populus deltoides*	crops (7)	laboratory bioassay, pot culture	leaves leaf	[118]
*Quercus coccifera*	crops (5)	laboratory bioassays	leaf and soil extracts	[119]
*Q. leucotrichophora*	*Quercus leucotrichophora*	laboratory bioassay, pot culture	bark, leaf, bark and leaf litter extracts	[120]
*Rhamnus cathartica*	herbs (4)	greenhouse, field	leaves and fruits	[121]
*R. cathartica*	herbs (3), trees (2)	pot culture	leaves and roots	[122]
*R. cathartica*	crops (1)	laboratory bioassay	fruits, leaf, root and bark extracts	[123]
*Rhododendron formosanum*	*Lactuca sativa*, crops (2), grasses (2), weed (1)	laboratory bioassays	flower, leaf and litter extracts	[124]
*R. formosanum*	*Lactuca sativa*	laboratory bioassays	allelochemical	[125]
*Robinia pseudo-acacia*	*Lactuca sativa*, crops (2), weeds (2), herbs (2)	laboratory bioassay	leaf extracts	[126]
*Tectona grandis*	crops (1), weeds (2)	laboratory bioassay	leaf extracts	[127]
*T. grandis*	*Lactuca sativa*, crops (4)	laboratory bioassay	allelochemicals	[128]
*T. grandis, Aleurites fordii, Gliricidia sepium, Maytenus buxifolia*	*Lactuca sativa*, crops (4)	laboratory bioassay	allelochemicals	[129]
*Tipuana tipu*	*Lactuca sativa*	laboratory bioassays	root, stem, leaf, flower and pod essential oils	[130]
*Ulmus pumila*	grasses (3)	laboratory bioassay, pot culture	leaf litter extracts	[131]
*Ziziphus spinachristi*	crops (4)	laboratory bioassay	leaf extracts	[132]

^a^ Test species are combined in crops, grass and weed groups. Number of species in each group are presented in parentheses. Full species names are given for the standard test species, as well as for the species, which autotoxicity was evaluated.

**Table 2:** Allelopathy of gymnosperm species.

Allelopathic Species	Test Species ^a^	Analysis Type	Compounds	Reference
*Araucaria angustifolia*	*Lactuca sativa*	laboratory bioassay	needle extracts	[135]
*Cunninghamia lancealata*	*Cunninghamia lancealata*	laboratory bioassay, pot culture	stump-roots extracts, stump-roots	[136]
*C. lancealata*	*Cunninghamia lancealata*	laboratory bioassay	leaf and root extracts, rhizosphere soil	[137]
*C. lancealata*	*Cunninghamia lancealata*	laboratory bioassay	root extracts	[138]
*C. lancealata*	*Cunninghamia lancealata*	laboratory bioassay	allelochemicals	[139]
*C. lancealata*	*Cunninghamia lancealata*	pot culture	leaf and plastic litter	[140]
*Ginkgo biloba*	*Lactuca sativa*, crop (1), grass (1), weed (1)	laboratory bioassay	leaf extracts, allelochemical	[141]
*Juniperus ashei*	grass (1)	sandwich method, field	leaf and litter leachate	[142]
*Latix gmelini*	tree (1)	pot culture	root, bark, branch and leaf extracts	[143]
*Picea schrenkiana*	*Picea schrenkiana, Latuca sativa*, crops (5)	laboratory bioassay	allelochemicals	[144]
*P. schrenkiana*	*Picea schrenkiana*	laboratory bioassay	needle extracts, allelochemicals	[145]
*Pinus densiflora*	crops (1), weeds (1)	laboratory bioassay	allelochemicals	[146]
*P. densiflora*	*Latuca sativa*, crops (2), weeds (3)	laboratory bioassay	allelochemicals	[147]
*Pinus halepensis*	*Lemna minor*, weeds (3)	laboratory bioassay, pot culture	needles, needle extracts	[148]
*P. halepensis*	*Pinus halepensis*	field	needle leachates	[149]
*P. halepensis*	*Lactuca sativa*, herb (1)	laboratory bioassay	root and needle extracts	[150]
*P. halepensis*	*Pinus halepensis*	laboratory bioassay	needle and roots extracts, litter	[151]
*P. halepensis*	*Pinus halepensis, Lactuca sativa*, herb (1)	laboratory bioassay	needle and roots extracts	[152]
*P. halepensis*	grasses (12)	laboratory bioassay	needle extracts, soil rhizosphere	[153]
*P. pinea*	weeds (3)	laboratory bioassay	essential oils	[154]
*P. roxburghii*	herb (1)	laboratory bioassay, pot culture	needles and bark extracts, needle litter	[155]
*P. thunbergii, P. tabuliformis, P. koraiensis*	*Pinus thunbergii, Pinus tabuliformis, Pinus koraiensis*	field	needle leachates	[156]
*Taxus baccata*	*Taxus baccata*	field	needls	[157]
*T. baccata*	crops (2)	laboratory bioassay	aril, leaf and bark extracts	[158]
*Thuja plicata, T. occidentalis, Abies amabilis, A. balsamea, A. grandis, A. lasiocarpa, Tsuga canadensis, T. mertensiana, T. heterophylla*	*Arabidopsis thaliana*	laboratory bioassay	extracts of resin vesicles from seeds	[159]
*Wollemia nobilis*	crops (1), grass (1)	laboratory bioassay	leaf extract	[160]

^a^ Test species are combined in crops, grass and weed groups. Number of species in each group are presented in parentheses. Full species names are given for the standard test species, as well as for the species, which autotoxicity was evaluated.
